# Schwann cells ER-associated degradation contributes to myelin maintenance in adult nerves and limits demyelination in CMT1B mice

**DOI:** 10.1371/journal.pgen.1008069

**Published:** 2019-04-17

**Authors:** Vera G. Volpi, Cinzia Ferri, Ilaria Fregno, Ubaldo Del Carro, Francesca Bianchi, Cristina Scapin, Emanuela Pettinato, Tatiana Solda, M. Laura Feltri, Maurizio Molinari, Lawrence Wrabetz, Maurizio D’Antonio

**Affiliations:** 1 Division of Genetics and Cell Biology, IRCCS San Raffaele Scientific Institute, Milan, Italy; 2 Instuitute for Research in Biomedicine, Faculty of Biomedical Sciences, Università della Svizzera italiana (USI), Bellinzona, Switzerland; 3 Department of Biology, Swiss Federal Institute of Technology, Zurich, Switzerland; 4 Institute of Experimental Neurology (INSPE), Division of Neuroscience, IRCCS San Raffaele Scientific Institute, Milan, Italy; 5 Hunter James Kelly Research Institute, University at Buffalo, Buffalo, New York, United States of America; 6 Department of Neurology, Jacobs School of Medicine and Biomedical Sciences, University at Buffalo, Buffalo, New York, United States of America; 7 Department of Biochemistry, Jacobs School of Medicine and Biomedical Sciences, University at Buffalo, Buffalo, New York, United States of America; 8 School of Life Sciences, École Polytechnique Fédérale de Lausanne, Lausanne, Switzerland; The Jackson Laboratory, UNITED STATES

## Abstract

In the peripheral nervous system (PNS) myelinating Schwann cells synthesize large amounts of myelin protein zero (P0) glycoprotein, an abundant component of peripheral nerve myelin. In humans, mutations in P0 cause the demyelinating Charcot-Marie-Tooth 1B (CMT1B) neuropathy, one of the most diffused genetic disorders of the PNS. We previously showed that several mutations, such as the deletion of serine 63 (P0-S63del), result in misfolding and accumulation of P0 in the endoplasmic reticulum (ER), with activation of the unfolded protein response (UPR). In addition, we observed that S63del mouse nerves display the upregulation of many ER-associated degradation (ERAD) genes, suggesting a possible involvement of this pathway in the clearance of the mutant P0. In ERAD in fact, misfolded proteins are dislocated from the ER and targeted for proteasomal degradation. Taking advantage of inducible cells that express the ER retained P0, here we show that the P0-S63del glycoprotein is degraded via ERAD. Moreover, we provide strong evidence that the Schwann cell-specific ablation of the ERAD factor Derlin-2 in S63del nerves exacerbates both the myelin defects and the UPR *in vivo*, unveiling a protective role for ERAD in CMT1B neuropathy. We also found that lack of Derlin-2 affects adult myelin maintenance in normal nerves, without compromising their development, pinpointing ERAD as a previously unrecognized player in preserving Schwann cells homeostasis in adulthood. Finally, we provide evidence that treatment of S63del peripheral nerve cultures with N-Acetyl-D-Glucosamine (GlcNAc), known to enhance protein quality control pathways in *C*.*elegans*, ameliorates S63del nerve myelination *ex vivo*. Overall, our study suggests that potentiating adaptive ER quality control pathways might represent an appealing strategy to treat both conformational and age-related PNS disorders.

## Author summary

Charcot-Marie-Tooth neuropathies are a large family of peripheral nerve disorders, showing extensive clinical and genetic heterogeneity. Although strong advances have been made in the identification of genes and mutations involved, effective therapies are still lacking. Intracellular retention of abnormal proteins has been recently suggested as one of the pathogenetic events that might underlie several conformational neuropathies. To limit the toxic effects of accumulated mutant proteins, cells have developed efficient protein quality control systems aimed at optimizing both protein folding and degradation. Here we show that ER-associated degradation limits Schwann cells stress and myelin defects caused by the accumulation of a mutant myelin protein into the ER. In addition, we also describe for the first time the importance of Schwann cells ERAD in preserving myelin integrity in adult nerves, showing that genetic ERAD impairment leads to a late onset, motor-predominant, peripheral neuropathy *in vivo*. Effort in the design of strategies that potentiate ERAD and ER quality controls is therefore highly desirable.

## Introduction

In the peripheral nervous system (PNS), Schwann cells discontinuously myelinate axons to promote the fast saltatory conduction of the nerve impulses. During myelin biogenesis, large amounts of both lipids and proteins are produced. After the completion of myelination, the maintenance of proper myelin homeostasis is essential for preserving axon functionality [1,2,3]. Mutations in myelin proteins, such as myelin protein zero (P0), cause different forms of Charcot-Marie-Tooth (CMT) neuropathy with mild-to-severe peripheral nerves dysfunctions [4,5]. In particular, the deletion of serine 63 in the extracellular domain of P0 (P0-S63del) results in a CMT1B neuropathy characterized by developmental hypomyelination followed by demyelination, with formation of onion bulbs-like structures [6,7]. Wild type (WT) P0, a type I transmembrane protein, is normally synthesized on ER-bound ribosomes, N-glycosylated at asparagine 122 and delivered to myelin via the secretory pathway [8,9,10]. The mutant P0-S63del is instead misfolded and retained in the ER, where it elicits a chronic ER stress that activates a dose-dependent unfolded protein response (UPR) [7,11,12]. The UPR, a cellular response aimed at rebalancing ER homeostasis, relies on the activation of signaling pathways which attenuate global protein synthesis and stimulate ER quality control (ERQC) systems, such as folding capacities and ER-associated degradation (ERAD) [13]. In ERAD of glycoproteins, the misfolded substrates are demannosylated, recognized by mannose-specific lectins and directed to multi-protein channels, the dislocons, that retrotranslocate them to the cytosol for proteasomal degradation [14,15]. An involvement of the ERAD pathway in the clearance of the misfolded P0-S63del protein was suggested by the observation that genes codifying for factors of the ERAD/proteasome system are globally upregulated in S63del mouse nerves [11,16]. Here we show that P0-S63del glycoprotein is degraded via ERAD *in vitro* and that the Schwann cell-specific ablation of the dislocon component Derlin-2 causes ERAD impairment and worsens the CMT1B phenotype in S63del mice. Furthermore, we show that ablation of Derlin-2 from WT Schwann cells results in a late-onset motor-predominant peripheral neuropathy. Thus, an efficient ERAD is not only protective in the S63del-CMT1B disorder, but is also important to preserve adult myelin integrity by maintaining ER homeostasis in Schwann cells. Compounds that enhance protein quality control pathways such as ERAD might therefore represent a new therapeutic avenue for ER stress- and age-related neuropathies.

## Results

### The P0-S63del protein is a target of ERAD

**Fig 1:**
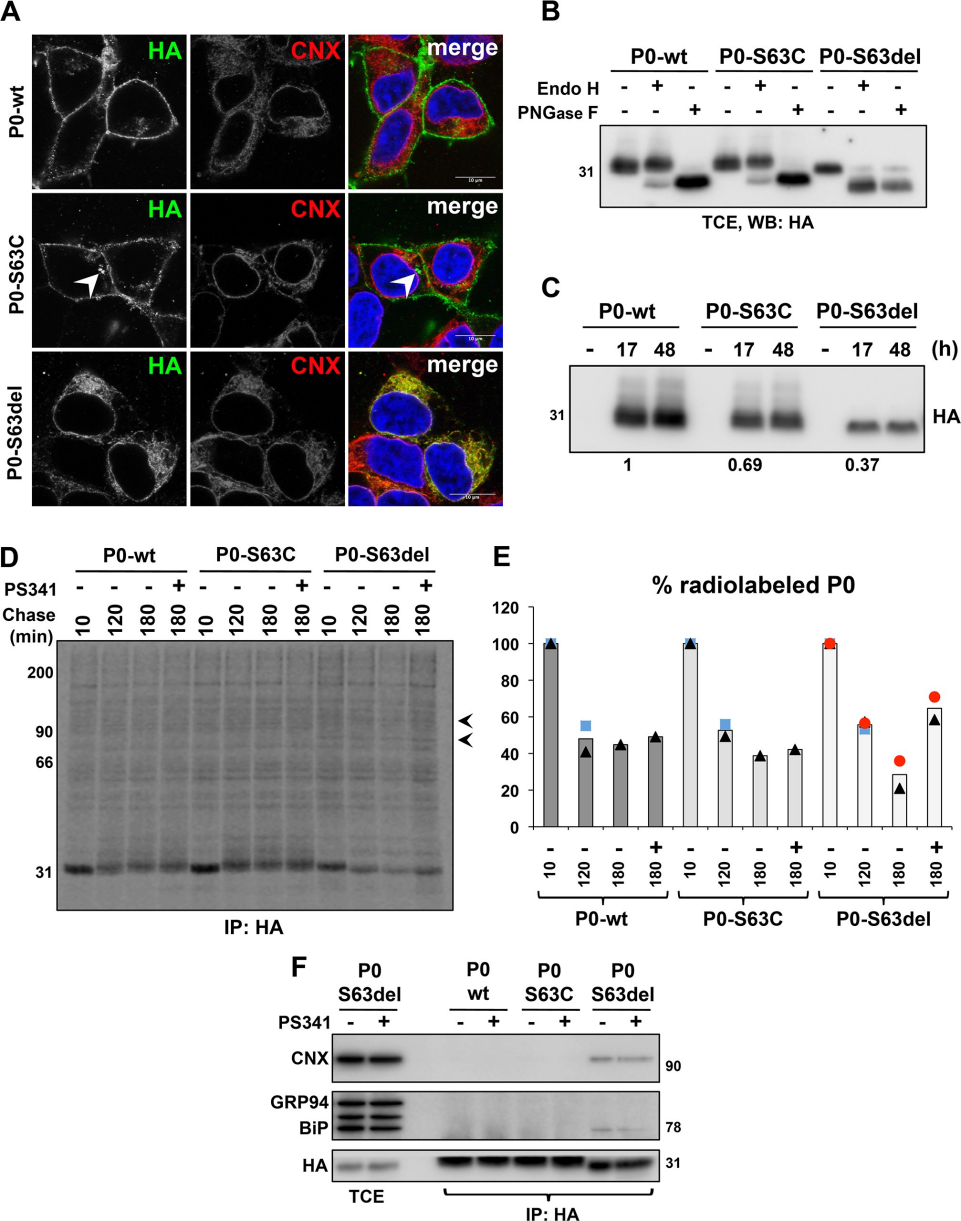
P0-S63del protein is degraded via ERAD. (A) Immunofluorescence staining on inducible HEK293 cells expressing P0-wt, P0-S63C or P0-S63del variants after 17 hrs of induction with 100 ng/ml tetracycline. Anti-HA antibody recognizes P0 (green), whereas anti-Calnexin (CNX) antibody (red) stains the ER; cells nuclei are visualized with DAPI (blue). White arrowhead points at aggregate-like intracellular structures formed specifically by P0-S63C variant. Scale bar, 10μm. (B) EndoH/PNGaseF assays on lysates from induced HEK293 cells, followed by Western blot against the HA-tagged P0s. (C) Steady state expression of P0 variants in cells either mock-treated or induced for 17 and 48 hr with 100 ng/ml tetracycline. One of two independent blots is shown. Quantification relative to WT is shown below the panel. Samples normalized for cell number. (D) Pulse-chase experiments on HEK293 cells induced for 17 hr. Cells were pulsed with [^35^S]-methionine/cysteine for 10 min and chased for 10 min, 120 min, 180 min or 180 min with PS341. P0 proteins were immunoprecipitated with anti-HA antibody and separated in SDS-PAGE under reducing conditions. Arrowheads indicate two bands that specifically co-immunoprecipitate with the P0-S63del variant. (E) Quantification of (D). Symbols represent the values obtained in the different replicates, whereas columns indicate the mean. Samples normalized for cell number; n = 1–3 replicates per condition. (F) Co-immunoprecipitation of HA-tagged P0s followed by Western blot against either BiP, detected with the anti-KDEL antibody, or CNX; induced HEK293 cells were treated for 3 hr with/without the proteasome inhibitor PS341 before lysis. (TCE, total cell extract).

Transcriptomic analysis of S63del mouse nerves, which are characterized by a chronic UPR [7,12], revealed the upregulation of several ERAD genes (S1A Fig) [11]. Accordingly, qRT-PCR and Western blot experiments performed on post-natal day 28 (P28) sciatic nerve samples confirmed the induction of a selection of ERAD factors at both the mRNA and protein levels (S1B and S1C Fig) [11]. This increase appeared to occur specifically in S63del Schwann cells, as suggested by immunofluorescence staining for the ERAD dislocation factor Derlin-2 on teased nerve fibers (S1D Fig). Co-immunoprecipitation experiments performed on P28 sciatic nerve lysates showed that Derlin-2 interacted with P0 in WT nerves and that this interaction was increased in S63del nerves, where also Derlin-1 appeared to interact with P0 (S1E, S1F and S1G Fig). These data and the observation that P0-S63del glycoprotein, but not wild-type P0, is polyubiquitinated [16] suggested that the ERAD/proteasome system might be involved in the degradation of the mutant P0-S63del *in vivo*. Several limitations, however, hamper the possibility to directly verify this hypothesis in nerves. In S63del Schwann cells both the wild type P0 and the mutant P0-S63del proteins are abundantly co-expressed [7] and, currently, there are no specific antibodies that discriminate between the two forms [16]. Thus, to test our hypothesis, we generated stable, tetracycline inducible HEK293 cells that express HA-tagged versions of either wild type P0 (P0-wt), P0-S63del or P0-S63C proteins. The latter was used as a control since, despite carrying a mutation in the same amino acid, the P0-S63C glycoprotein exits the ER-Golgi stacks and reaches the myelin sheath [7,17]. Immunofluorescence analysis confirmed that also in this *in vitro* system P0-wt and P0-S63C reached the cell surface, whereas P0-S63del protein is retained in the ER, as shown by its co-localization with the ER marker Calnexin (CNX) (Fig 1A). Accordingly, endoglycosidase H (EndoH) assay showed that the N-glycan portion of P0-S63del protein was largely sensitive to EndoH cleavage, indicative of a folding defect that blocks the protein into the ER. The control proteins were instead mostly EndoH resistant, since they assume a mature and transport-competent conformation (Fig 1B). As expected, all these proteins were sensitive to treatment with peptide-N-glycosidase F (PNGaseF), an enzyme that removes almost all types of N-glycan independently of the intracellular localization of the proteins (Fig 1B) [18]. Western blot analysis performed 17 and 48 hrs after induction with tetracycline showed that the steady state levels of both P0-S63C and, to greater extent, P0-S63del proteins were reduced as compared to P0-wt (Fig 1C). However, pulse-chase experiments did not detect any gross difference in their rate of synthesis (S2A and S2B Fig). These observations suggest that the mutant P0s, and particularly the P0-S63del protein, may have reduced intracellular stability and faster degradation rate as compared to P0-wt. To test whether the ERAD/proteasome system degrades the two P0 mutants, we treated the induced cells with the proteasome inhibitor PS341 and performed pulse-chase analysis followed by immunoprecipitation and SDS-PAGE (Fig 1D and 1E). Western blot against ubiquitin confirmed that PS341 blocked proteasome activity, resulting in a global accumulation of polyubiqitinated proteins (S2C Fig.) Differently from P0-wt and P0-S63C, P0-S63del protein displayed higher electrophoretic mobility in pulse-chase experiments (Fig 1D), typical of mannose trimming that occurs when misfolded proteins are retained in the ER before degradation [19]. Importantly, P0-S63del protein showed faster degradation rates as compared to controls and accumulated following proteasome inhibition (Fig 1D and 1E). We also noted that two additional polypeptides with MW around 90 and 70 KDa appeared to co-immunoprecipitate with the P0-S63del protein, but not with the other two P0 variants (see arrowheads in Fig 1D and S2A Fig). Based on their crucial role as ER retention factors for unstructured glycoproteins, we hypothesized that these two proteins could be CNX and binding immunoglobulin protein (BiP). To verify this, induced cells were treated with PS341 and subjected to immunoprecipitation against P0, followed by Western blot analysis to specifically reveal BiP and CNX (Fig 1F). As expected, these chaperones were substantially detectable only in P0-S63del immunocomplexes (Fig 1F) and, in addition, pulse-chase followed by double co-immunoprecipitation experiments, which reveal only stable interactions, confirmed these results (S2D and S2E Fig). Altogether these data corroborate our hypothesis that the ERAD pathway participates in the clearance of the ER-retained P0-S63del glycoprotein, limiting its toxicity.

### The ERAD factor Derlin-2 is not required for myelin formation and remyelination, but its ablation worsens developmental hypomyelination in S63del CMT1B mice

Based on these observations, we hypothesized a protective role for ERAD in the S63del CMT1B neuropathy. To test this, we impaired ERAD in peripheral nerves via the Schwann cell-specific ablation of Derlin-2, a well characterized ERAD factor and UPR target gene [20,21,22], whose deletion was previously shown to cause defective ER dislocation *in vivo* [23]. Importantly, Derlin-2 protein is upregulated in S63del Schwann cells and it appears to interact with P0 (S1 Fig). We crossed Der2^fl/fl^ mice [23] with P0Cre mice [24] to obtain P0Cre//Der2^fl/fl^ or ^fl/+^ mice. These mice were then bred with S63del//Der2^fl/+^ animals to generate S63del//P0Cre//Der2^fl/fl^ mice (S63del//Der2^SCKO^), P0Cre//Der2^fl/fl^ mice (Der2^SCKO^) and the respective P0Cre-negative controls. To test the efficiency and specificity of P0Cre-mediated recombination, we performed PCR reactions on genomic DNA extracted from sciatic nerves and several other tissues (S3A and S3B Fig). In P5 sciatic nerves the recombined Der2^KO^ (600bp) band was specifically detected in all P0Cre-positive samples, but not in P0Cre negative control nerves, as expected (S3A Fig). Moreover, in P21 Der2^SCKO^ mice, the recombination band appeared only in sciatic nerves and not in other tissues, with the exception for a faint Der2^KO^ band visible in skeletal muscles, possibly because of the presence of nerve terminals (S3B Fig). Accordingly, Derlin-2 mRNA and protein levels were consistently decreased in Der2^SCKO^ and S63del//Der2^SCKO^ nerves as compared to the respective controls (S3C–S3E Fig). To assess ERAD impairment in Derlin-2 knockout Schwann cells, we measured the levels of the lectin chaperone osteosarcoma amplified 9 (OS9) and of inositol-requiring enzyme 1α (IRE1α), two endogenous ERAD substrates that get stabilized in several ERAD deficient tissues [23,25,26,27,28]. In both Der2^SCKO^ and S63del//Der2^SCKO^ nerves we found remarkably high amounts of OS9 protein, but minimally changed mRNA levels (S3F–S3H Fig), indicating that, also in peripheral nerves, the lack of Derlin-2 prevalently leads to OS9 protein stabilization because of a less efficient degradation. Similarly, IRE1α protein was strongly increased in Der2^SCKO^ and S63del//Der2^SCKO^ nerves (S3I and S3J Fig). A mild stabilization of endogenous ERAD substrates was also observed in S63del controls (S3F–S3H, S3I and S3J Fig), possibly because of the diminished proteasomal efficiency in these nerves [16].

**Fig 2:**
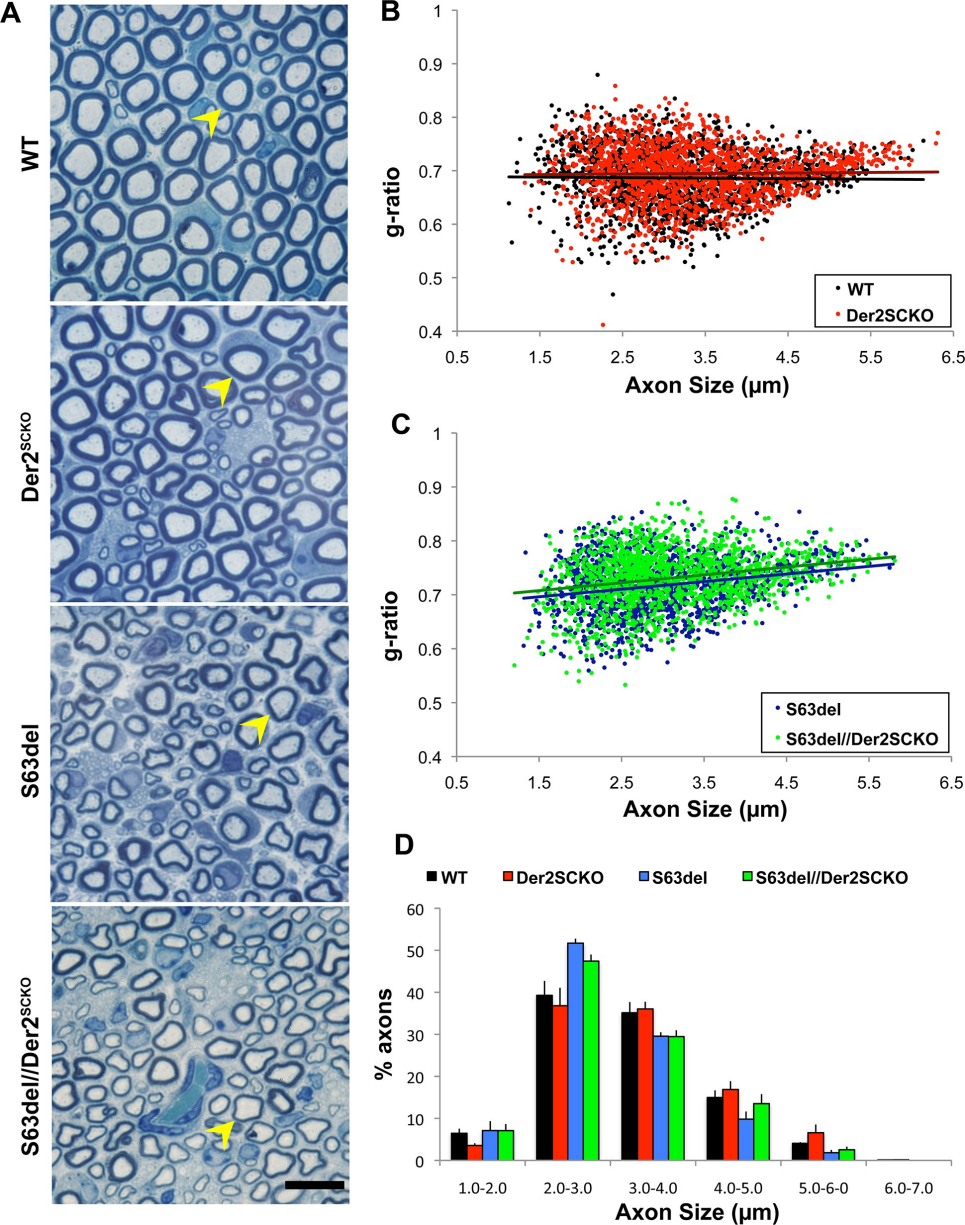
Morphology of Der2^SCKO^ and S63del//Der2^SCKO^ sciatic nerves at P28. **(**A) Transverse semithin sections from WT, Der2^SCKO^, S63del and S63del//Der2^SCKO^ P28 sciatic nerves. Arrowheads indicate fibers with similar diameter for myelin thickness comparison. n = 5–6 mice/genotype. Scale bar, 10μm; 100x magnification. (B-C) Morphometric g-ratio (axon diameter/fiber diameter) analysis on P28 sciatic nerve sections. Scatter-plots of g-ratio distribution with trend lines are shown. ~1500–1700 fibers/genotype; n = 3 nerves per genotype. (D) Graph representing the frequency of myelinated axons (as percentage of all myelinated axons) per axon size interval (μm) at P28.

Given the highly secretory nature of myelinating Schwann cells, we reasoned that the impairment of ERAD might have detrimental effects on myelination. To test this hypothesis, we collected Der2^SCKO^ sciatic nerves at P5, P15 and P28 and evaluated their morphology. At all these time points, Der2^SCKO^ nerves did not display any gross myelin abnormality (Fig 2A and S4A Fig). At P28, myelin thickness was normal as measured by morphometric g-ratio analysis on semithin sections (mean g-ratio: Der2^SCKO^ 0.69±0.001; WT 0.68±0.003, n.s.; Fig 2B), and the axon size distribution was unchanged as compared to WT controls (Fig 2D). To assess whether Derlin-2 is instead required for remyelination, we performed sciatic nerve crush experiments on Der2^SCKO^ mice and looked at the extent of remyelination 45 days after injury (T45). Morphological analysis performed on semithin sections did not reveal any significant difference in the extent of remyelination, with comparable numbers of remyelinated and degenerated fibers in Der2^SCKO^ and WT injured controls (S4B–S4D Fig). Moreover, g-ratio analysis performed on T45 EM sections confirmed that myelin reached a similar thickness in both Der2^SCKO^ and WT remyelinated nerves (S4E and S4F Fig), suggesting that Derlin-2 is not required for correct remyelination after injury.

Notably however, ablation of Derlin-2 in S63del nerves worsened the developmental hypomyelination (Fig 2A), with a significant increase in the mean g-ratio at P28 (S63del//Der2^SCKO^ 0.73±0.003; S63del 0.71±0.001, P = 0.015; Fig 2C), but no effects on the axon size distribution (Fig 2D). Detailed EM analysis confirmed equal myelination in Der2^SCKO^ and WT developing nerves, and increased hypomyelination in S63del//Der2^SCKO^ nerves as compared to S63del control, without significant alterations in Schwann cell morphology (S5A and S5B Fig). Finally, Derlin-2 deletion did not impact on Schwann cell number in P21 sciatic nerve sections (S5C and S5D Fig). In fact, whereas S63del nerves showed supernumerary Schwann cells as compared to WT, as previously reported [12], the number of cells nuclei was basically unaltered in both Der2^SCKO^ and S63del//Der2^SCKO^ as compared to the respective WT and S63del controls (S5C and S5D Fig).

Altogether these data indicate that Derlin-2 is neither required for normal developmental myelination nor for remyelination after injury, but it appears to be protective in early stages of the CMT1B neuropathy.

### Derlin-2 ablation perturbs ER homeostasis in developing Schwann cells and exacerbates the UPR in S63del-CMT1B nerves

**Fig 3:**
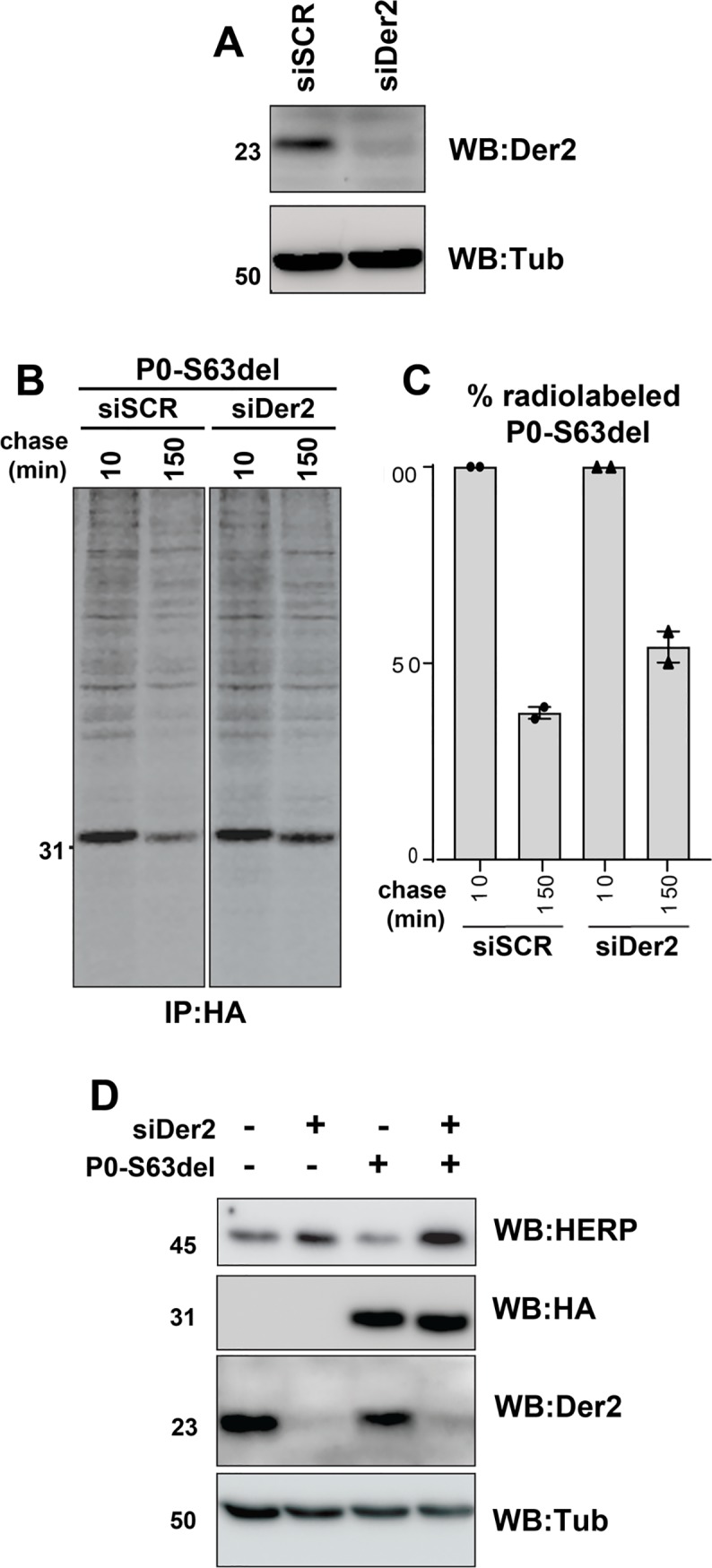
Depletion of Derlin-2 leads to P0-S63del accumulation and ER-stress activation. (A) Western blot analysis for Derlin-2 on HEK293 cells transfected with scramble (SCR) or Der2 siRNA. One representative blot of two is shown. (B) Pulse chase experiment on P0-S63del expressing HEK293 cells induced for 18 hr with 100 ng/ml tetracycline after Der2 silencing. Cells were pulsed with [^35^S]-methionine/cysteine for 10 min and chased for 10 min and 150 min. One representative gel of two is shown. (C) Quantification of (B). (D) Western blot for the ER stress marker HERP on HEK293 cells expressing the HA-tagged P0-S63del protein, after Derlin-2 silencing. One representative blot of two is shown.

We have previously shown that an increase in P0-S63del protein expression determines higher UPR induction and a more severe neuropathy in transgenic S63del mice [7,12]. We reasoned that, since ERAD appears to be involved in the degradation of the P0-S63del glycoprotein, its impairment should stabilize the misfolded P0 in the ER and increase the levels of stress, leading to the worsening of the phenotype observed in S63del//Der2^SCKO^ mice. To test this hypothesis, we first checked the effects of siRNA-driven Derlin-2 depletion on the stabilization of P0-S63del protein in HEK293 cells. We confirmed the efficient silencing of Derlin-2 by Western blot (Fig 3A) and then performed pulse-chase experiments, followed by immunoprecipitation and SDS-PAGE, to monitor the levels of radiolabeled P0-S63del protein (Fig 3B and 3C). After 150 min of chase, Der2 knocked-down cells displayed 20% higher levels of radiolabeled P0 as compared to control cells, confirming that the depletion of Derlin-2 leads to P0-S63del protein accumulation (Fig 3B and 3C). In addition, we also found that the silencing of Derlin-2, even in the absence of P0-S63del expression, activates a stress response, as shown by increased homocysteine-induced endoplasmic reticulum protein (HERP) protein levels [29,30], whose induction is further intensified following the accumulation of P0-S63del protein (Fig 3D), in agreement with increased expression of HERP in P28 S63del nerves (S1A and S1B Fig).

**Fig 4:**
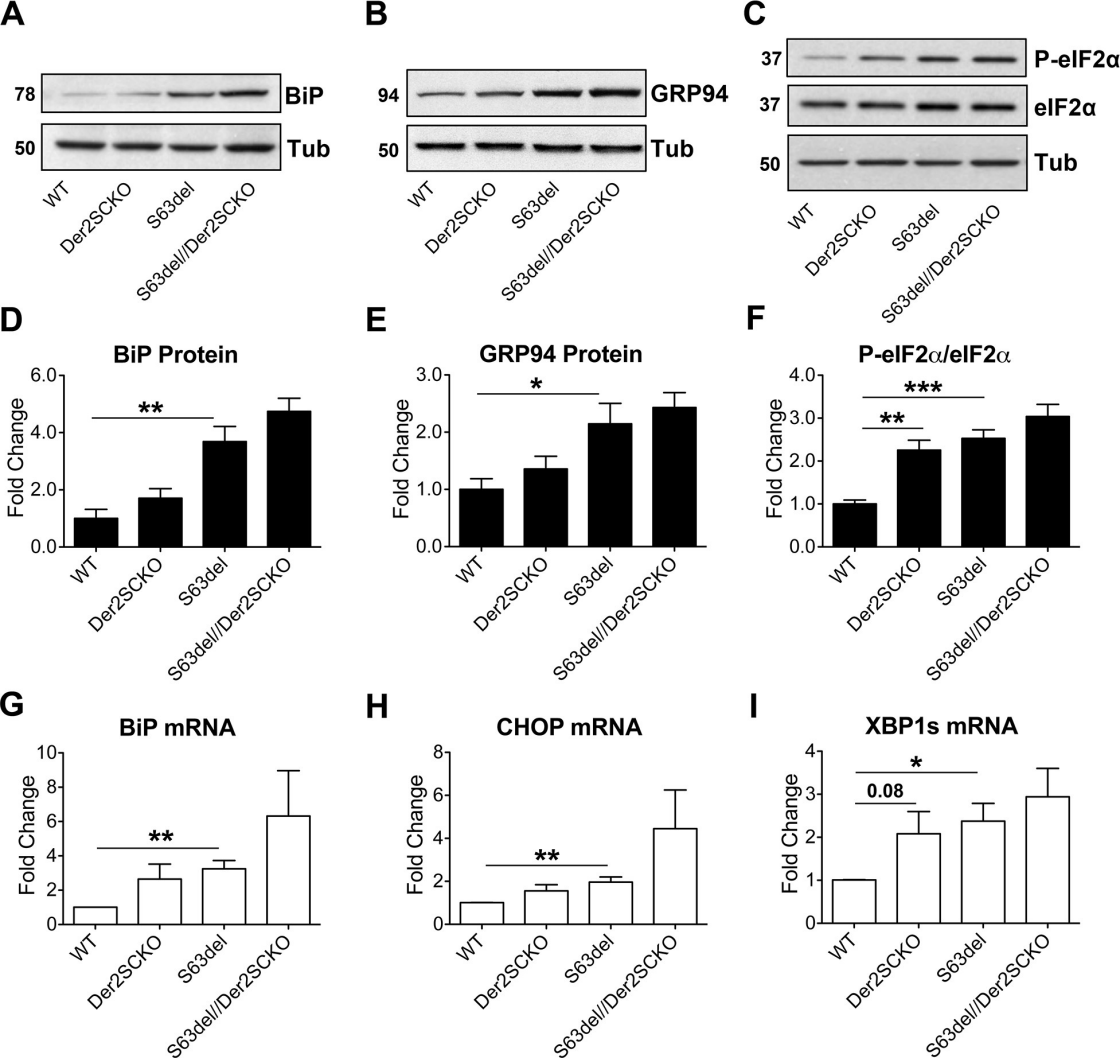
Measurement of ER stress/UPR levels in mutant sciatic nerves at P28. (A-C) Western blot analysis for the ER stress/UPR markers BiP, GRP94 and P-eIF2α on P28 sciatic nerve lysates; β-Tubulin was used as loading control. One representative blot of four is shown. (D-F) Protein levels of BiP, GRP94 and P-eIF2α as measured by densitometric analysis. (G-I) qRT-PCR analysis on P28 sciatic nerve extracts for BiP, CHOP and spliced Xbp1; WT animals were used as the reference group. n = 4–5 RT from independent pools of three nerves per genotype. Error bars, SEM; *P < 0,05, **P < 0,01, ***P < 0,001 by unpaired, 2-tails, Student’s *t* test.

Next, as a measure of ER-retained P0-S63del levels *in vivo*, we checked the expression of ER stress/UPR markers such as BiP, C/EBP-Homologous Protein (CHOP), Glucose Regulated Protein 94 (GRP94), phosphorylated eukaryotic Initiation Factor 2 α (P-eIF2α) and spliced X-box Binding Protein 1 (Xbp1s) in sciatic nerves at P28. As hypothesized, S63del//Der2^SCKO^ nerves showed a consistent trend towards higher levels for all the markers analyzed as compared to S63del nerves, suggesting higher P0-S63del levels in the ER (Fig 4A–4I). Despite overall normal Schwann cells development and myelination (Fig 2), and in line with what seen in the HEK293 cells following Derlin-2 silencing, also Der2^SCKO^ sciatic nerves showed signs of modest ER stress and UPR induction as compared to WT controls (Fig 4A–4I). These observations resemble what was previously reported in Derlin-2 deficient hepatocytes and B-cells, in which both the secretory function and cell development appeared normal despite mild UPR activation elicited by lack of Derlin-2 [23].

### ERAD impairment alters myelin morphology and functionality in both WT and S63del adult sciatic nerves

**Fig 5:**
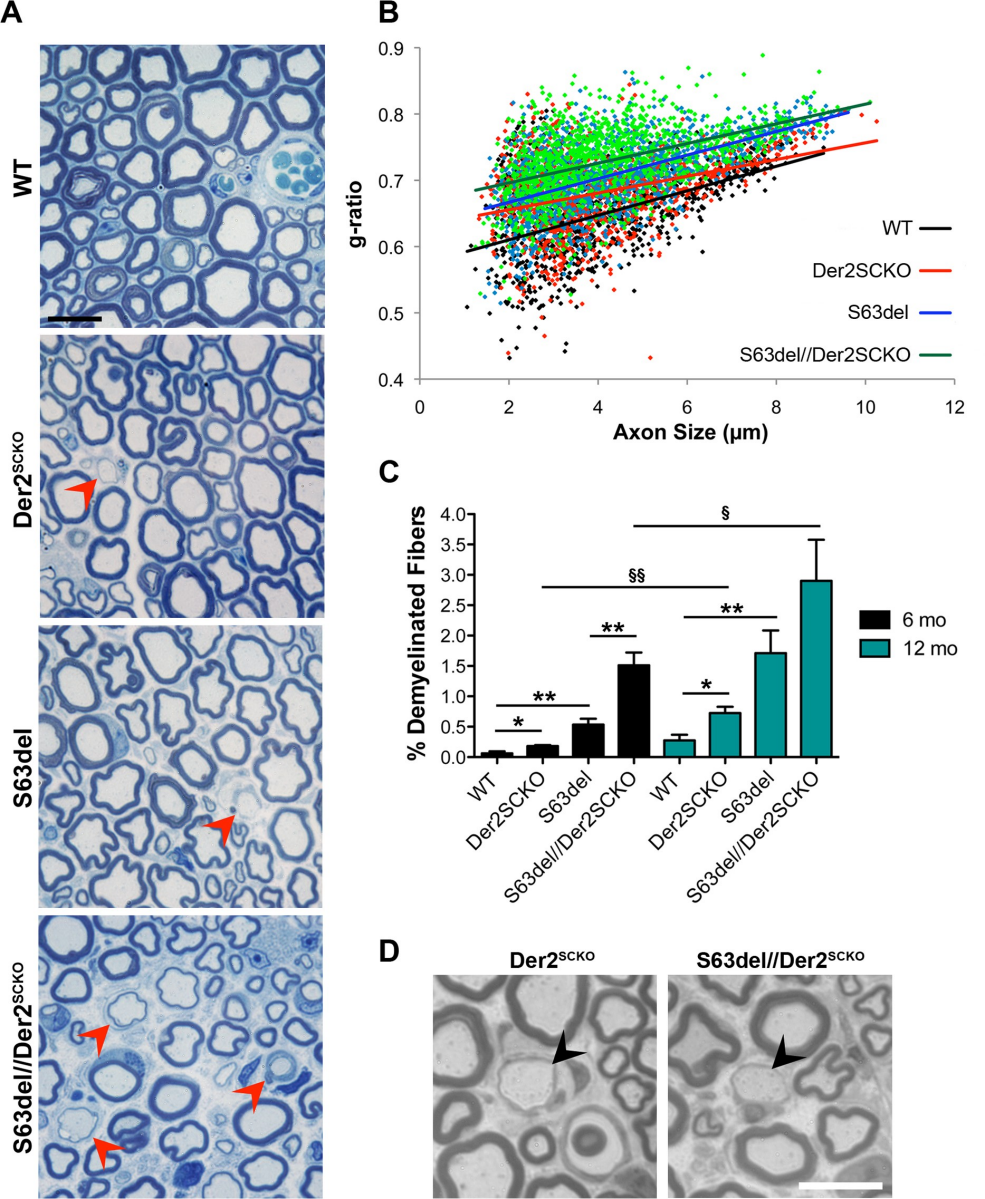
Morphology of Der2^SCKO^ and S63del//Der2^SCKO^ adult sciatic nerves. (A) Transverse semithin sections from WT, Der2^SCKO^, S63del and S63del//Der2^SCKO^ sciatic nerves at 12 mo. Arrowheads indicate demyelinating/remyelinating fibers. n = 4–5 mice/genotype. Scale bar, 10**μ**m; 100x magnification. (B) Morphometric g-ratio analysis performed on sciatic nerve semithin sections at 6 mo. Scatter-plot of g-ratio distribution with trend lines is reported; ~800–1200 fibers/genotype. n = 3–4 nerves per genotype. (C) Number of demyelinated fibers in 6 and 12 mo sciatic nerves (expressed as a percentage of total myelinated fibers per sciatic nerve field). 8–10 non-overlapping fields per nerve were analyzed from n = 3–5 nerves per genotype. Error bars, SEM; * and § P < 0,05, ** and §§ P < 0,01 by Student’s *t* test. (D) Transverse semithin sections of 6 mo Der2^SCKO^ and S63del//Der2^SCKO^ motor fascicles of sciatic nerves in which large onion bulbs and naked axons are visible (arrowheads). Scale bar, 10**μ**m.

**Fig 6:**
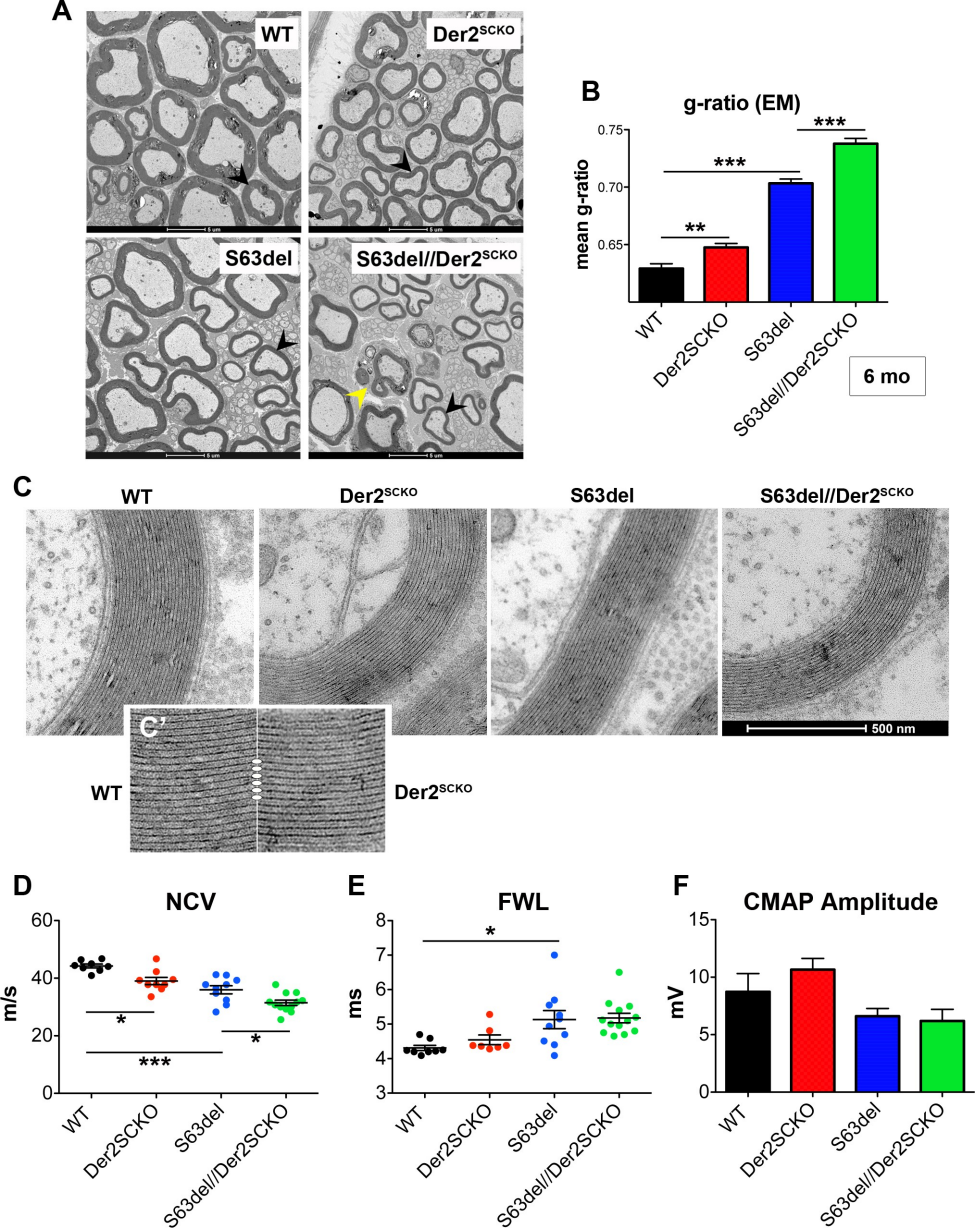
Ultrastructural and neurophysiological analyses in adult Der2^SCKO^ and S63del//Der2^SCKO^ nerves. (A) EM images from WT, Der2^SCKO^, S63del and S63del//Der2^SCKO^ sciatic nerves at 6 mo. Black arrowheads show axons of similar caliber for myelin thickness comparison. Yellow arrowhead shows a Schwann cell with ongoing demyelination. Scale bar, 5**μ**m (B) Mean g-ratio quantification (WT 0.63± 0.004; Der2^SCKO^ 0.65±0.003; S63del 0.70±0.004; S63del//Der2^SCKO^ 0.74±0.005); n = 50–70 fibers per nerve, from three nerves per genotype. Error bars, SEM; **P < 0,01, ***P < 0,001 by one-way ANOVA with Tukey’s post hoc test. (C-C’) Ultrastructural analysis of myelin in 6 mo WT, Der2^SCKO^, S63del and S63del//Der2^SCKO^ shows proper myelin compaction with normal periodicity in Der2^SCKO^ as compared to WT (C’). (D) Measurement of nerve conduction velocities (NCV; m/s), (E) F wave latencies (FWL; ms) and (F) compound muscle action potential (CMAP) amplitudes (mV) on 6 mo sciatic nerves. In (E), only detectable FWLs are reported in the graph; 30% of FWLs in Der2^SCKO^ and 7% in S63del//Der2^SCKO^ nerves, versus 0% in WT and S63del controls, were totally absent. n = 8–12 nerves per genotype. Error bars, SEM; *P < 0,05, **P < 0,01 by one-way ANOVA with Tukey’s post hoc test.

In S63del mice demyelination occurs predominantly in adulthood [7,12]. We thus analyzed the effects of ERAD impairment also in 6–12 months old (mo) sciatic nerves. In S63del//Der2^SCKO^ nerves, the myelin sheath appeared thinner as compared to S63del controls (mean g-ratio: S63del//Der2^SCKO^ 0.73±0.007; S63del 0.71±0.004, P = 0.043; Fig 5A and 5B), consistently with what already observed at P28. Rather unexpectedly, adult Der2^SCKO^ sciatic nerves showed reduced myelin thickness as well (Der2^SCKO^ 0.68±0.002; WT 0.65±0.009, P = 0.032), suggestive of mild nerve pathology (Fig 5A and 5B). In addition, at both 6 and 12 mo, S63del//Der2^SCKO^ sciatic nerves showed a strong increase in the number of demyelinated fibers as compared to S63del nerves (Fig 5A and 5C), and features of progressive demyelination appeared also in Der2^SCKO^ mice (Fig 5A and 5C). Of note, in both S63del//Der2^SCKO^ and Der2^SCKO^ mice, large naked axons and onion bulbs predominantly appeared within the motor areas of sciatic nerves, composed of fascicles of medium-large caliber fibers (Fig 5A and 5D). Quantitative EM analysis confirmed the hypomyelination in S63del//Der2^SCKO^ nerves as compared to S63del nerves, and in Der2^SCKO^ nerves as compared to WT (Fig 6A and 6B). Ultrastructural analysis showed that myelin periodicity was grossly unaltered in Der2^SCKO^ as compared to WT, indicating that the reduction in myelin thickness was likely due to a reduced number of myelin wraps rather than a defect in myelin compaction (Fig 6C–6C’).

Next, we evaluated nerve functionality by measuring nerve conduction velocity (NCV), F-wave latency (FWL) and compound muscle action potential (CMAP) amplitude. In line with the observed phenotypes, NCVs were significantly reduced in both Der2^SCKO^ and S63del//Der2^SCKO^ nerves as compared to the respective controls (WT 44.24±0.678; Der2^SCKO^ 39.01±1.243; S63del = 35.98±1.384; S63del//Der2^SCKO^31.45±0.947; Fig 6D). FWL, which was higher in S63del nerves as compared to WT as expected, was slightly increased by the ablation of Derlin-2 and, remarkably, 30% of the FWLs in Der2^SCKO^ and 7% in S63del//Der2^SCKO^ nerves were absent (Fig 6E). Derlin-2 ablation in both WT and S63del backgrounds, instead, did not significantly alter CMAP amplitudes (Fig 6F). Altogether these data indicate that a functional ERAD contributes to myelin maintenance in normal adult nerves and protects Schwann cells from demyelination in CMT1B neuropathy with activated UPR.

### ERAD impairment causes severe demyelination of motor-predominant nerves

**Fig 7:**
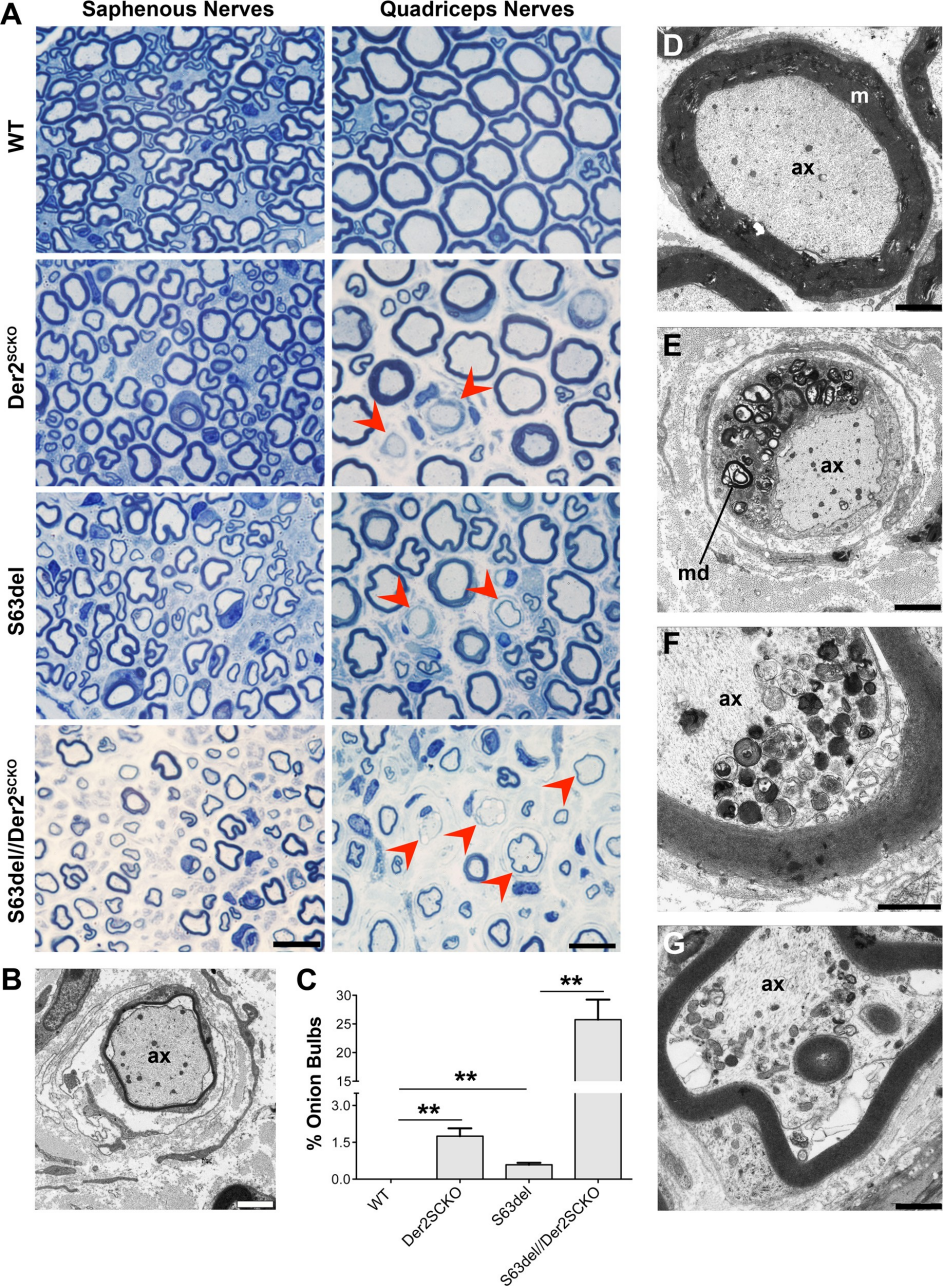
Morphology of Der2^SCKO^ and S63del//Der2^SCKO^ adult quadriceps and saphenous nerves. (A) Transverse semithin sections of saphenous and quadriceps nerves from WT, Der2^SCKO^, S63del and S63del//Der2^SCKO^ at 12 mo. Red arrowheads indicate onion bulbs. n = 4–5 nerves/genotype. Scale bar, 10**μ**m; 100x magnification. (B) Image of an onion bulb by EM analysis on 12 mo S63del//Der2^SCKO^ quadriceps nerves. Scale bar, 2**μ**m; ax, axon. (C) Number of onion bulbs in 12 mo quadriceps nerve (expressed as a percentage of total myelinated fibers in the whole nerve); n = 3–4 nerves/genotype. Error bars, SEM; *P < 0,05, **P < 0,01 by Student’s *t* test. (D-G) EM analysis on 12 mo quadriceps and saphenous nerves. In (D), a normal fiber from WT quadriceps nerve is shown as control. Panel (E) shows a demyelinating fiber from S63del//Der2^SCKO^ quadriceps nerves in which the cell surrounding the axon is full of myelin debris (md). In (F) and (G), signs of axonal degeneration detected in S63del//Der2^SCKO^ quadriceps and saphenous nerves, respectively. (D-E) scale bar, 2**μ**m; (F-G), scale bar, 1**μ**m.

The morphological analysis outlined above suggested that onion bulbs were more frequent in the motor fascicles rather than in the sensory ones of sciatic nerves in both Der2^SCKO^ and S63del//Der2^SCKO^ mice (Fig 5A and 5D). Thus, we reasoned that the motor component of nerves could be more susceptible to ERAD perturbation as compared to the sensory component. To test this hypothesis, we took advantage of femoral nerves, in which a motor-predominant branch (quadriceps nerves) and a sensory branch (saphenous nerve) can be analyzed separately. Der2^SCKO^ quadriceps nerves showed more pronounced onion bulbs formation as compared to sciatic nerves (compare Fig 7A with Fig 5A), whereas Der2^SCKO^ saphenous nerves appeared grossly normal (Fig 7A). In S63del//Der2^SCKO^ mice, instead, both quadriceps and saphenous nerves were severely compromised, although with some remarkable differences. In fact S63del//Der2^SCKO^ quadriceps nerves, but not saphenous nerves, showed extensive onion bulbs formation and signs of ongoing demyelination (Fig 7A–7C and 7E) as compared to the control nerve (Fig 7A and 7D), similar to what observed in high P0-S63del overexpressor (S63del-H) [7]. Despite this difference however, both nerves displayed signs of axonal degeneration (Fig 7F and 7G). Overall these data indicate that ablation of Derlin-2 in WT mice causes an age-related, motor-predominant, demyelinating neuropathy, suggesting that motor nerves are more sensitive to ERAD impairment as compared to sensory nerves. In S63del mice instead, ERAD impairment aggravates the CMT1B disease phenotype in all nerves analyzed, with motor fibers presenting the most severe demyelination.

### Derlin-2 ablation leads to a proteostatic failure in adult WT nerves and exacerbates the UPR in CMT1B nerves

**Fig 8:**
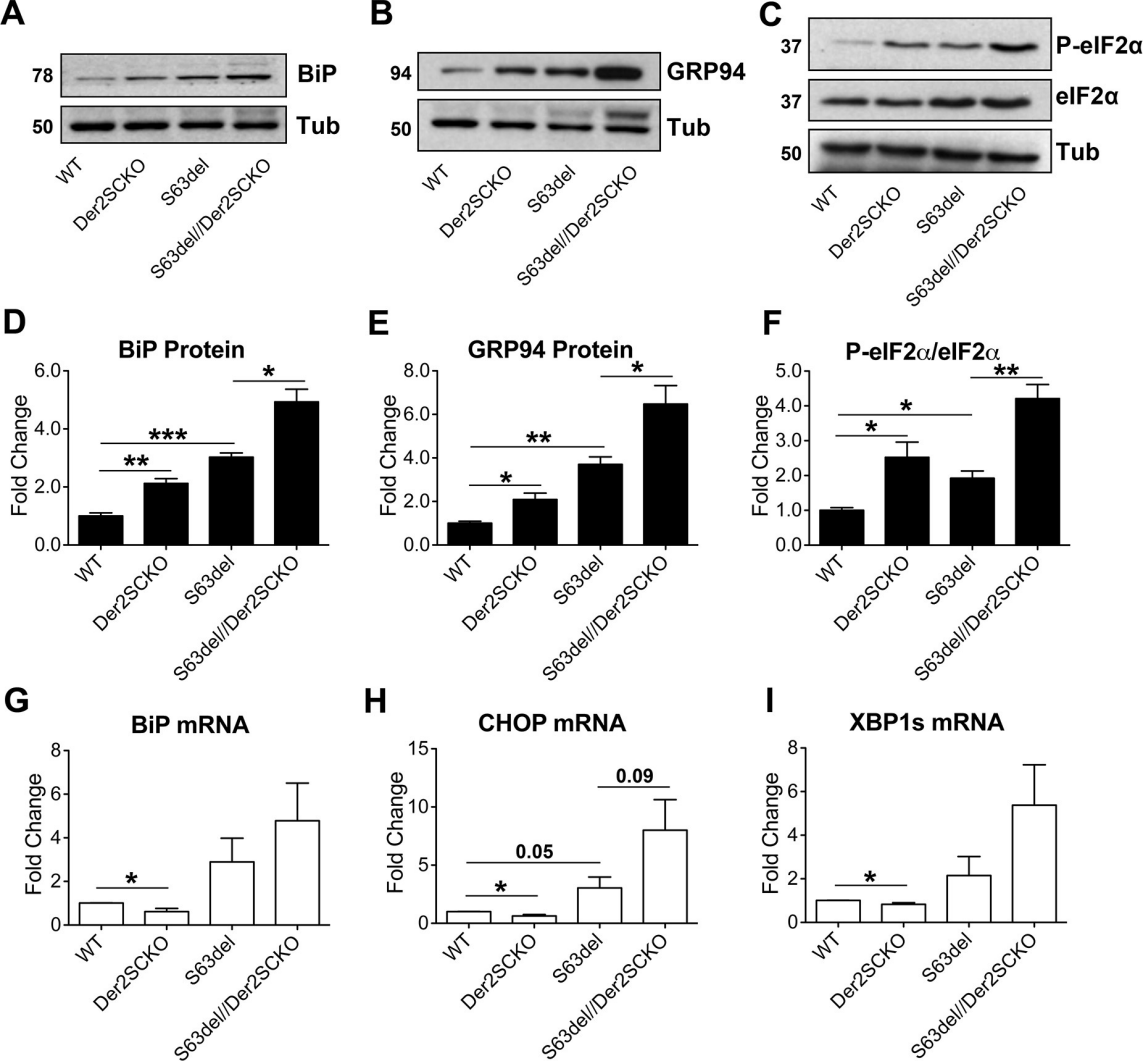
Measurement of ER stress/UPR levels in adult sciatic nerves. (A-C) Western blot analysis for the ER stress/UPR markers BiP, GRP94 and P-eIF2α on 6 mo sciatic nerve lysates; β-Tubulin was used as loading control. One representative blot of three is shown. (D-E-F) Protein levels of BiP, GRP94 and P-eIF2α as measured by densitometric analysis. (G-H-I) qRT-PCR analysis for BiP, CHOP and spliced Xbp1 on 6 mo sciatic nerve extracts; the WT was used as the reference group. n = 5–6 independent RT per genotype. Error bars, SEM; *P<0,05, **P<0,01, ***P<0,001 by unpaired, 2-tails, Student’s *t* test.

The progressive and severe worsening of the neuropathy in S63del//Der2^SCKO^ mice and the appearance of a phenotype in adult Der2^SCKO^ nerves prompted us to analyze stress responses also at this stage. In 6 mo nerves ERAD impairment due to Derlin-2 ablation was still evident, as shown by the increased levels of OS9 and IRE1α protein in both Der2^SCKO^ and S63del//Der2^SCKO^ sciatic (S6A–S6C Fig) and quadriceps (S6D–S6F Fig) nerves. Western blot and qRT-PCR experiments showed a strong increase in both ER stress levels and UPR activation in adult S63del//Der2^SCKO^ nerves as compared to S63del controls (Fig 8A–8I and S7A–S7E Fig). Conversely, Der2^SCKO^ sciatic nerves, which showed significant increase of BiP and GRP94 protein levels (Fig 8A, 8B, 8D and 8E), did not display canonical activation of the UPR branches. In fact, BiP and Xbp1s mRNAs (targets of ATF6 and IRE1 respectively) were not induced (Fig 8G–8I) and only P-eIF2α levels appeared to be increased as compared to WT (Fig 8C and 8F), although the phosphorylation of eIF2α was uncoupled from CHOP induction (Fig 8H). In Der2^SCKO^ quadriceps nerves, where demyelination was more pronounced (Fig 7), we instead detected a trend towards an increase of CHOP (S7E Fig), correlating with previous observations suggesting that CHOP activation underlies demyelination in peripheral nerves [12].

Taken together these results suggest a complex scenario in which in S63del//Der2^SCKO^ nerves ERAD impairment determines an increase in the ER retention of P0-S63del protein augmenting ER stress/UPR, progressively worsening the neuropathic phenotype. In Der2^SCKO^ sciatic nerves instead, the perturbation of ERAD transiently activates a mild UPR during development, most likely favouring cell fitness [31]. The UPR however fails to be maintained into adulthood, possibly determining a proteostatic failure that may underlie myelin degeneration during aging.

### Treatments with N-acetyl-D-glucosamine (GlcNAc) ameliorate S63del myelination *ex vivo*

**Fig 9:**
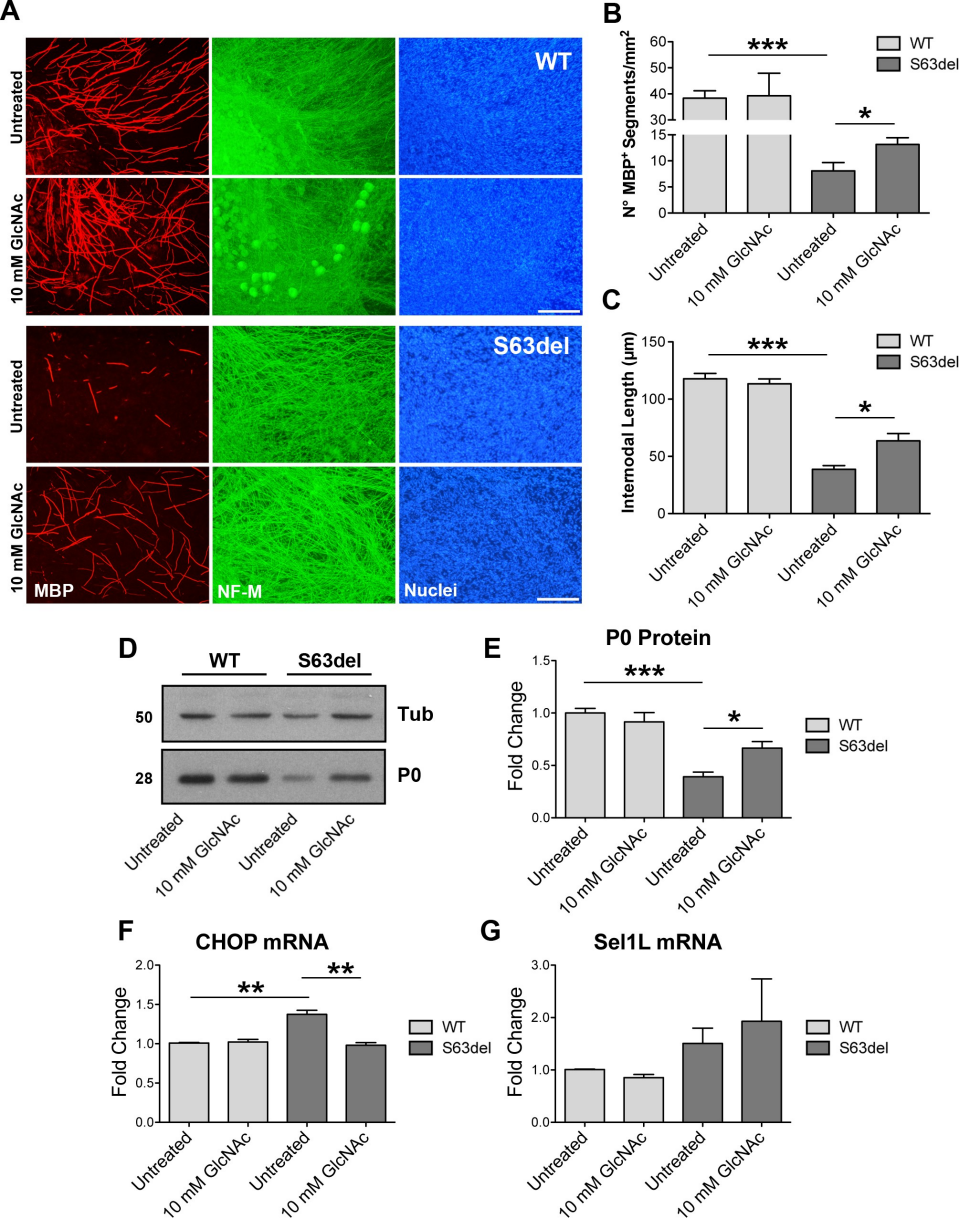
GlcNAc treatments ameliorate myelination in S63del DRG explants. (A) Myelinating DRG explants were dissected from E13.5 WT and S63del littermate embryos. Treatment with 10 mM GlcNAc was performed for 2-weeks, in parallel to the induction of myelination with ascorbic acid. Myelinated internodes were detected with antibodies against myelin basic protein (MBP; red). Neurofilament (NF)-M (green) staining marks the axons; nuclei are visualized with Hoechst (blue). Scale bar, 100μm. (B-C) Graphs showing the number of MBP^+^ internodes/mm^2^ (B) and the internodal length (C), respectively. 5–12 DRG explants/condition/dissection; n = 4 independent dissections. Error bars, SEM. *P <0,05 by unpaired, 2 tails, Student’s *t* test. (D) Western blot analysis for P0 on lysates from DRG explants treated with 10 mM GlcNAc as in (A). One representative blot of three is shown. (E) Protein levels of P0 as measured by densitometric analysis. (F-G) qRT-PCR analysis for CHOP and Sel1L on extracts form DRG explants treated with 10 mM GlcNAc for 3 weeks. n = 3 independent embryos per genotype, 7–8 DRG/embryo/condition. Error bars, SEM; *P<0,05, **P<0,01, ***P<0,001 by unpaired, 2-tails, Student’s *t* test.

Overall our data suggest that in CMT1B neuropathy ERAD is an adaptive ERQC pathway that limits the toxic effects of the misfolded P0. Potentiating the adaptive ERQC systems might therefore represent an appealing approach for the treatment of misfolded-protein diseases. In this respect, it has been shown that the stimulation of the hexosamine biosynthetic pathway (HBP), which generates intermediates for N- and O-glycosylation of proteins [32], promotes stress resistance, relief from proteotoxicity and lifespan extension in *C*.*elegans* by globally enhancing protein degradation systems, including ERAD [33]. Thus, we hypothesized that treatments with the HBP intermediate N-acetyl-glucosamine (GlcNAc) could improve S63del myelination. To test this hypothesis, both WT and S63del dorsal-root-ganglia (DRG) explants were allowed undergoing myelination *ex vivo* for 2 weeks in presence or absence of GlcNAc. These treatments appeared well tolerated, as suggested by normal myelination in treated WT DRG explants (Fig 9A–9C). Treatment with GlcNAc significantly improved myelination in S63del DRG explants as measured by the increased number and length of MBP^+^ internodes (Fig 9A–9C) and increased P0 protein, as measured by WB (Fig 9D and 9E). This improvement was accompanied by a significant reduction in stress levels as measured by qRT-PCR for CHOP (Fig 9F), even though we could detect only a small, not significant increase in the mRNA for the ERAD component Sel1L (Fig 9G), that in *C*. *elegans* appears the main ERAD target of the HBP pathway [33]. Further studies will be required to test whether and how the stimulation of the HBP pathway can alleviate nerve pathology *in vivo*.

## Discussion

Protein misfolding and the activation of ER-stress are emerging as a common pathomechanism in many neurodegenerative disorders, including CMT neuropathies [34,35,36]. The removal of potentially toxic proteins within the cell is one of the adaptive functions of the main cellular degradation pathways, such as the ERAD/proteasome system and autophagy. Here we show that Schwann cell ERAD is protective in an ER stress-related demyelinating neuropathy and contributes to healthy aging of peripheral nerves.

S63del nerves are characterized by ER-retention of the mutant P0-S63del protein, activation of a UPR and a strong induction of genes encoding for components of the ERAD/proteasome system [11]. Recently it has been shown that P0-S63del protein is polyubiquitinated [16], and we found that the ERAD factors Derlins interact with P0 *in vivo*, in particular in S63del nerves. These observations suggested that the ERAD/proteasome system might exert a central role in the clearance of the misfolded P0. Taking advantage of inducible cells, we provided evidence that the misfolded P0-S63del protein stably interacts with BiP and CNX and is rapidly degraded via ERAD. Both P0-wt and P0-S63C instead exit the ER, possibly binding the lectin chaperone CNX only transiently [37]. The different trafficking of P0-wt and P0-S63del proteins might explain why VerPlank et al. detected an impairment of the proteasome function in S63del nerves, but not in nerves that overexpress wild type P0 [16]. Interestingly, the P0-S63C mutant, that did not appear to be degraded via ERAD, displayed reduced stability and tendency to form intracellular aggregates (Fig 1A). Other quality control pathways might therefore participate in its disposal, for example autophagy or peripheral quality controls at the plasma membrane, as those involved in the clearance of the mutant CFTR [38]. In light of this, it would be interesting to analyze how the cellular degradation systems remove the different P0 mutants and whether the correct modulation of these pathways can alleviate the associated neuropathies.

### ERAD is adaptive in the CMT1B neuropathy

The S63del-CMT1B neuropathy is characterized by developmental hypomyelination, followed by demyelination with onion bulbs formation and compromised nerve functionality [6,7]. Deletion of Derlin-2 from S63del Schwann cells exacerbates both the early and late features of the neuropathy, indicating that ERAD is protective, most likely because of its role in degrading the misfolded P0-S63del protein, as our *in vitro* data suggest. In S63del//Der2^SCKO^ nerves, in fact, ER stress and UPR levels are higher as compared to S63del controls. This is in line with the idea of increased P0-S63del accumulation in the ER, and accordingly, the phenotype of S63del//Der2^SCKO^ nerves closely recapitulates what happens in S63del-H mice [7,12]. In S63del//Der2^SCKO^ mice, quadriceps and saphenous nerves manifest a dramatic exacerbation of the disease, whereas sciatic nerves appear only moderately worsened. The reason for this difference is currently unknown. It would be intriguing to assess whether these nerves differ in their inflammatory response or susceptibility to inflammation, since a similar disease feature is often observed in sporadic inflammatory neuropathies [39,40].

Finally, the observation that deletion of Derlin-2 causes *per se* a late onset demyelinating neuropathy (see below) and that its deletion synergizes with the P0-S63del mutation worsening the CMT1B phenotype, may suggest that also ERAD genes, alongside integral nodal component such as Nrcam and Scn8a [41,42,43], could be among those genetic modifiers that influence the large phenotypic variability of many forms of CMTs.

### Enhancement of adaptive protein degradation as a therapeutic approach

The number of CMT1B-causing mutations that activate a UPR is constantly increasing [44,45] and, in addition, other mutant myelin proteins of both PNS and CNS are characterized by ER retention and/or proteasomal degradation, such as PMP22 [46,47,48], connexin-32 [49], MAG [50] and PLP mutants [51,52]. This enlarges the spectrum of myelinating disorders for which treatments based on pharmacological modulation of ERQC systems and/or UPR might be a promising therapy [36]. Here we show that absence of Derlin-2 dramatically exacerbates the S63del-CMT1B neuropathy, pointing at the enhancement of ERAD as a potential strategy to treat the disease. In line with this, overexpression of Derlin-2 was shown to protect renal podocytes from apoptosis caused by ER dysfunction [53] and upregulation of EDEM was shown to preserve against ER proteotoxicity and age-related physiological decline in *Drosophila* [54]. In addition, pharmacological inhibition of USP14, a deubiquitinating enzyme, was shown to enhance the proteasomal degradation of some disease-associated misfolded proteins in cultured cells [55,56] and to increase the rate of general proteolysis in S63del sciatic nerves *ex vivo* [16]. However, USP14 inhibitors proved extremely toxic when used in myelinating DRG explants, hampering the possibility to test their effects on S63del myelination. Still, here we show that treatments of S63del DRG explants with GlcNAc, a HBP metabolite known to enhance ERAD, the proteasome and autophagy in *C*.*elegans* [33], ameliorated the extent of S63del myelination and reduced the levels of ER-stress. It should be noted that GlcNAc administration might also increase lipid synthesis and accumulation [57], which are known to be downregulated in S63del mice [11]. Further studies are needed to assess whether and how administration of GlcNAc can alleviate the disease in mouse models of conformational neuropathies.

### Der2^SCKO^ mice manifest a late onset neuropathy

Although the proteasome regulates the levels of wild type PMP22 [58,59] and of some PMP22 mutants [48], the involvement of ERAD in myelination has been poorly investigated and its role in the degradation of ER-retained myelin proteins is only currently emerging [50,60]. Of note, ablation of BiP and CNX, ER retention factors upstream of ERAD, has been shown to cause myelin abnormalities in both the PNS and the CNS [61,62,63]. Here we show that Schwann cell-specific deletion of Derlin-2 impairs ERAD in WT nerves, but does not affect developmental myelination or remyelination after nerve injury. Despite this, P28 Der2^SCKO^ sciatic nerves display moderate ER stress and transient UPR induction. This suggests that low levels of stress can be tolerated by the developing Schwann cells, in which ERQC pathways and stress responses might be sufficiently tonic to cope with the reduced protein degradation efficiency, similarly to B-cell and hepatocytes [23]. Aged Der2^SCKO^ mice instead develop a demyelinating neuropathy, indicating that an efficient ERAD becomes important in adulthood to preserve myelin integrity. In humans, peripheral neuropathies associated to aging are highly diffuse but their pathogenetic mechanisms are still largely unknown [64,65]. Our data, together with the observation that aged tissues, including peripheral nerves, encounter a natural decline in expression and performance of general proteostatic networks [11,66,67,68,69], make it tempting to speculate that ERQC failure could be one of the factors that contribute to the onset of age-related neuropathies. Indeed, in line with the idea of a decline in the efficiency of stress responses, in adult Der2^SCKO^ sciatic nerves the UPR fails to be activated. Only the phosphorylation of eIF2α remains sustained although its downstream target CHOP is not significantly induced. This could suggest that PERK, upstream of the P-eIF2α/CHOP arm of the UPR, might not be the only kinase responsible for eIF2α phosphorylation in Der2^SCKO^ nerves. Several works indicated that the GCN2/P-eIF2α branch of the integrated stress response (ISR) [70], mainly activated upon amino acid starvation and UV irradiation, can be, in some cases, a less potent inducer of CHOP as compared to PERK [71,72]. Moreover, proteasome inhibition was shown to activate the ISR because of reduced amino acid recycling [73]. Thus, it is reasonable to imagine that impaired ER dislocation may limit the amount of proteins destined for proteasomal degradation, rendering the recycling of amino acids less efficient and favoring the activation of the GCN2/P-eIF2α branch in Derlin-2 deficient Schwann cells.

Our data also suggest that motor fibers appear to rely on an efficient ERAD to greater extent as compared to sensory fibers, although the reason for this difference is unknown. A possibility is that different subpopulations of myelinating Schwann cells might exist, capable of re-adjusting their phenotypes depending on the type of fiber they myelinate and, thus, showing different susceptibility to ERQC failure. This would be in agreement with what was suggested by Höke et al. and Brushart et al., who showed that myelinating Schwann cells express distinct phenotypes at least for what concerns the pattern of growth factors production [74] and central-peripheral location [75]. Alternatively, these differences might be intrinsic to each nerve and depend on its anatomy, origin, function and/or usage.

### Conclusions

Currently, effective therapies for CMT and age-related neuropathies are missing. Our data allow envisaging two, not mutually exclusive, approaches to target ERQC for therapeutic intervention in ER-stress related CMTs: the attenuation of known maladaptive arms of the UPR [11,76] and the enhancement of adaptive pathways, such as ERAD. Similarly, interventions aimed at modulating the proteostasis network may prove beneficial also for age-related neuropathies. Further efforts in the discovery of drugs able to potentiate ERQC pathways are therefore highly desirable.

## Materials and methods

### Ethics statement

All experiments involving animals were performed in accordance with Italian national regulations and covered by experimental protocols reviewed by local Institutional Animal Care and Use Committees. Approval number 359/2015-PR.

### Transgenic mice

S63del (S63del-L line), P0Cre and Derlin-2^fl/fl^ mice have been previously described [7,23,24]. S63del mice were maintained on FVB/N genetic background, whereas P0Cre and Derlin-2^fl/fl^ lines were maintained on C57/BL6N background (Charles River, Calco, Italy). Der2^SCKO^ (P0Cre//Der2^fl/fl^) and S63del//Der2^SCKO^ (S63del//P0Cre//Der2^fl/fl^) mice (FVB//C57BL6—F2 generation) were obtained by crossing S63del//Der2^fl/+^ (FVB//C57BL6—F1 generation) and P0Cre//Der2^fl/fl^ or ^fl/+^ mice (C57/BL6N). Age matched S63del (S63del//Der2^fl/fl^ or ^+/+^) and WT (Der2^fl/fl^ or ^+/+^) littermates were used as controls. For genotyping and evaluation of P0Cre-mediated recombination, genomic DNA was extracted from tail, sciatic nerve, skeletal muscle, brain, heart, spleen and kidney. All PCR products were stained with SYBR Safe DNA Gel Stain (Invitrogen), run in 2% agarose gels and detected with UVP GelDOC-It Imaging System. PCR protocols for genotyping of S63del and P0Cre mice were previously described [7,24]; Derlin-2 PCR primer sequences were: 5’-GGTTCATGCAGACAAACCATGATCGC-3’; 5’AGAGTGAAATGGCAGTTGGGTGTG-3’; 5’-GCTTTCACAAACCTGCAAGCTCCT-3’

### Myelinating Dorsal Root Ganglia (DRG) explants cultures

DRG explants were isolated from E13.5 embryos, seeded on rat collagen I-coated coverslips and maintained in culture as previously described [77]. Myelination was induced with 50μg/ml ascorbic acid (Sigma-Aldrich) added to culture medium. Treatments with N-acetyl-D-Glucosamine (GlcNAc; Sigma-Aldrich), dissolved in culture medium, were performed for 2 or 3 weeks in parallel to myelination induction. Culture medium was refreshed every two days. Samples were fixed and prepared for immunofluorescence and the average number and length of MBP^+^ internodes per field were measured with NIH Image-J software. 8 non-overlapping images per DRG were acquired with a Leica DM5000 microscope (10x and 20x objectives) equipped with a Leica DFC480 digital camera. At least 3 independent dissections were performed.

### Inducible cell lines

Inducible cell lines where generated using the Flp‐In T‐REx system (Invitrogen). HA-tagged P0-wt, P0-S63C and P0-S63del cDNAs were cloned in *Hin*d III ‐ *Eco*R V sites in the pCDNA5/FRT/T0 vector. The constructs were subjected to DNA sequencing to confirm the DNA preparations. To obtain the inducible cell lines, Flp‐In T‐REx HEK293 cells were co‐transfected with pCDNA5/FRT/T0 plasmids encoding the P0 variants and with a pOG44 construct that constitutively expresses the Flp recombinase. Cells were selected using medium supplemented with 150μg/ml hygromycin and 15μg/ml blasticidin. Flp‐In T‐REx HEK293 cells expressing the P0 proteins were cultured in DMEM supplemented with 10% FBS, 150μg/ml hygromycin and 15μg/ml blasticidin. Induction of P0s transgenes expression was obtained by adding 100ng/ml tetracycline to cell culture medium.

### Morphological and morphometric analyses of nerves

Nerves were freshly dissected, fixed in 2% glutaraldehyde in phosphate buffer, osmicated in 1% OsO_4_, alcohol dehydrated, infiltrated with propylene oxide and embedded in Epon. Transverse semithin sections and ultrathin sections were cut with an Ultracut microtome [11,78]. Semithin sections were stained with toluidine blue and acquired with a Leica DM5000 microscope equipped with a DFC480 digital camera, whereas ultrathin sections were stained with lead citrate and photographed with a Zeiss (Oberkochen, Germany) EM10 electron microscope. g-ratio (axon diameter/fiber diameter) was measured on semithin sections with semi-automated computer based morphometric analysis using Leica QWin V3 software [11]; four-six images per nerve were acquired with a 100x objective; ~800–2000 fibers per condition were measured. On EM images, g-ratio was measured using ImageJ software. 50–70 myelinated fibers from 10–12 images per animal were analyzed, from three mice per genotype. The number of demyelinated (naked) axons was counted blind to genotype on images acquired with a 100x objective from sciatic nerve semithin sections. The number of onion bulbs was measured on entire quadriceps nerves: single images were acquired with a 40x objective and nerves were reconstructed with Adobe-Photoshop CS4 (Adobe Systems, San Jose, CA). Three-five animals per genotype were used.

### Electrophysiology

Electrophysiological tests were performed using an EMG system (NeuroMep Micro, Neurosoft, Russia). Mice were anesthetized and placed under a heating lamp to maintain a constant body temperature. Monopolar needle electrodes were inserted subcutaneously to stimulate the tibial nerve at the ankle and, subsequently, the sciatic nerve at the sciatic notch; the cathode was placed close to the nerve and the anode was inserted proximally to the cathode. The stimulation consisted of single 100μs, 1Hz supramaximal pulses. The muscular response was recorded by inserting the active electrode into muscles in the middle of the paw and the reference electrode in the skin between the first and second digit. NCV (m/s), peak-to-peak CMAP amplitude (mV) and FWL (ms) were measured. FWL measurement was obtained by stimulating the tibial nerve at the ankle and recording the responses in the paw muscles, using the same pair of needle electrodes used for the nerve conduction study [79].

### Nerve Teasing and Cryosections

Nerves were freshly dissected, desheated in PBS and teased after 20 min of fixation with 4% paraformaldehyde (PFA). Nerve fibers were gently separated, let adhere onto slides and stored at -80°C until immunofluorescence. Images were acquired with Volocity Software at Perkin Elmer Ultraview ERS Confocal microscope with a 63x objective and processed with Adobe Photoshop CS4 (Adobe Systems, San Jose, CA). For cryosections preparation, sciatic nerves were immediately embedded in Killik cryostat embedding medium (Bio-Optica), frozen in liquid nitrogen and stored at -80°C until analysis.

### Antibodies

The following rabbit antibodies recognized PMP22 (1:10000; Abcam), GRP78/BiP (1:1000; Stressgene), Calnexin (1:2000; Sigma Aldrich; 1:3000 WB—1:500 IP -1:100 IF; kind gift by A. Helenius), OS9 (1:10000, Abcam), eIF2α and P-eIF2α (1:2000; Cell Signalling XP-Technology), Ubiquitin (1:2000, Dako). Rabbit anti-Derlin-1 and anti-Derlin-2 antibodies were received from H. L. Ploegh [23]. Rabbit antibody against HERP was a generous gift of K. Kokame. Rat antibodies recognized GRP94 (1:2000; Abcam) and MBP (1:5). Chicken monoclonal antibody recognized Neurofilament-M (1:1000; BioLegend). Mouse antibodies recognized ß-Tubulin (1:5000/1:10000; Sigma Aldrich), HA tag (1:500–1:100; Hybridoma 12CA5; Santa Cruz) and KDEL (1:200 IF, ENZO Life Sciences; 1:700–500 WB-IP, Stressgene).

### Immunofluorescence

HEK293 cells were plated on glass poly‐lysine coated coverslips. After 17 hr of induction, cells were fixed using 3,7% formaldehyde and blocked with 10% goat serum. Primary and secondary antibodies, diluted in 10% goat serum, were incubated for 2 hr and 30 min respectively. Microscopy images were collected using a laser scanning confocal microscope (Leica DI6000 microscope stand, SP5 scan head) equipped with a HCX PL APO CS 63X oil UV objective. DRG explants, sciatic nerve cryosections and teased nerve fibers were fixed for 15 min with 4% PFA and permeabilized with ice-cold methanol or 0.1% Triton-X100 (Sigma) in blocking solution. Samples were blocked in normal goat serum (NGS; Dako)/1% bovine serum albumine (BSA; Sigma)/PBS for 1 hr at room temperature (RT). Primary and secondary antibodies were diluted in 1% BSA. Primary antibodies were incubated 1 hr RT or overnight at 4°C, whereas secondary antibodies 45 min at RT in dark condition. Nuclei were marked using Hoechst or DAPI. Samples were mounted onto slides with Vectashield mounting medium (Vector Laboratories).

### RNA interference

For siRNA-based interference, Flp‐In T‐REx HEK293 cells expressing the P0-S63del protein were grown in DMEM supplemented with 10% FBS. Cells at 50% confluence were transfected with 50 pmol/dish siRNA duplex (hs_DERL2 FlexiTube siRNA Qiagen) using Lipofectamine 2000 according to the manufacturer’s instructions. 30 h after siRNA transfection, the expression of P0-S63del was induced by adding 100ng/ml tetracycline to cell culture medium. 48 h after siRNA transfection, cells were lysed or subjected to pulse-chase analysis as described below.

### Metabolic labeling and pulse-chase analysis

Induced cells were washed with PBS and incubated with starving medium (DMEM, 50mM Hepes, 1% Glutamax) for 10 min at 37°C. [^35^S]‐methionine/cysteine mix (SIGMA‐Aldrich) was directly added to a final concentration of 0.2mCi/ml and cells were pulsed for 10 min. Label medium was removed and cells were chased in DMEM supplemented with 5mM non‐labeled methionine/cysteine. Cells were washed with PBS containing 20mM N-ethyl-maleimide (NEM) for 1 min and then lysed with 2% CHAPS (Anatrace) in HEPES‐buffered saline (HBS), pH 6.8, supplemented with 20mM NEM and protease inhibitors for 20 min on ice. Supernatants were collected by centrifugation at 4°C/10000xg for 10 min, immunoprecipitated and subjected to SDS‐PAGE as described below. After exposure of the gels to autoradiography films (GE Healthcare, Fuji), films were scanned with the Typhoon FLA 9500 (Software Version 1.0).

### Immunoprecipitation of ectopically expressed proteins

For immunoprecipitation, cell lysates were incubated with protein-A beads (SIGMA, 1:10, w/v swollen in PBS) and the specific antibody. After 90 min, the immunocomplexes were washed with HBS, 0.5% CHAPS, pH 6.8. Beads were resuspended in sample buffer and denatured for 10 min at 65°C. Samples were subjected to SDS‐PAGE (see below).

### SDS PAGE and Western blot analysis

Peripheral nerves were dissected and frozen in liquid nitrogen. Frozen nerves were pulverized on dry ice and proteins were extracted in denaturing lysis buffer (Tris/HCl 50mM PH7.5, NaCl 150mM, EDTA 10mM, 2% SDS) containing protease inhibitor cocktail (PIC 100X roche), Na3VO4 and NaF. Total protein concentration was determined by BCA assay (Pierce) following manufacturer’s instructions. Equal amounts of proteins were separated by SDS-PAGE (Biorad) and gels were transferred onto nitrocellulose membrane (GE Healthcare). Membranes were blocked with 5% milk (milk powder/1x PBS-Tween 0.05%) and incubated with primary antibodies diluted in 5% Milk or 5% BSA/1x PBS-Tween 0.05% at 4°C overnight. HRP-conjugated antibodies were diluted in 5% Milk/1x PBS-Tween 0.05% and incubated 1 hr RT. Signals were detected by ECL method and autoradiography film (GE Healthcare) with Classic E.O.S. AGFA Developer Machine. Densitometric analysis was performed with NIH-Image-J software. For inducible cells, protein samples were prepared as described above and separated in SDS‐PAGE under reducing conditions after boiling in DTT‐containing sample buffer for 10 min at 65°C. Membranes were developed using the Luminata Forte ECL detection system (Millipore) and signals were detected with the ImageQuant LAS 4000 system in the standard acquisition mode (GE Healthcare Life Science). Bands were quantified using Multi Gauge Analysis tool (Fujifilm). The linearity of the detected signal range was ensured with appropriate loading controls.

### TaqMan quantitative polymerase chain reaction analysis

Total RNA was extracted with Trizol (Roche Diagnostic GmbH, Germany) and retrotranscribed as previously described [7]. TaqMan assays were performed following manufacturer’s instructions (TaqMan, PE Applied Biosystems Instruments) on an ABI PRISM 7700 sequence detection system (Applied Biosystems Instruments) [11,12]. Normalization was performed using 18S rRNA as reference gene. Target and reference genes PCR amplification were performed in separate tubes with Assay on Demand (Applied Biosystems Instruments): 18S assay, Hs99999901_s1; Ddit3/Chop assay, Mm00492097_m1; Xbp-1s assay, Mm03464496_m1; Hspa5/BiP assay, Mm00517691_m1; Derl3 assay, Mm00508292_m1; Derl2 assay, Mm01245788_m1; Derl1 assay, Mm00470296_g1; Sel1L assay, Mm01326442_m1; HRD1/SYVN1 assay, Mm00511995_m1; EDEM1 assay, Mm00551797_m1; OS9 assay, Mm00617153_m1; Herpud1 assay, Mm00445600_m1.

### Statistical analysis

Sample size was not predetermined with any statistical method, but our sample size is similar to that generally used in the field. Graphs and data were analyzed using GraphPad Prism Software and/or Microsoft Excel. Data show the mean ± Standard Error of Mean (SEM). Unpaired, 2 tails, Student’s *t* test or One-way ANOVA with Tukey’s post hoc test were used as specified in the figure legends; significance levels (P values) were marked on figures as follows: *P ≤ 0.05, **P ≤ 0.01, ***P ≤ 0.001; only comparisons between WT vs Der2^SCKO^, WT vs S63del and S63del vs S63del//Der2^SCKO^ groups are illustrated in all figures.

## Supporting information

S1 Fig ERAD factors induction and interaction with P0 in S63del nerves. (A) Expression of ERAD genes in P28 S63del nerves relative to WT as measured by microarray analysis [11]. (B) qRT-PCR for a selection of ERAD genes on P28 S63del sciatic nerve extracts. n = 5 RT from independent pools of nerves. Error bars, SEM.; *P < 0,05, **P < 0,01 by Student’s *t* test. (C) Western blot on P28 sciatic nerve lysates against the ERAD members Derlin-1 and -2; β-Tubulin was used as loading control. One of four independent blots is shown. (D) Immunofluorescence for Derlin-2 (green) on P21 teased nerve fibers. In red, KDEL staining marks the Schwann cell ER; in blue, Schwann cells nuclei are visualized with Hoechst staining. Scale bar, 10**μ**m. (E-F-G) Immunoprecipitation on WT and S63del sciatic nerve lysates with either anti-Derlin-1 (E) or anti-Derlin-2 (F) antibodies, followed by Western blot for P0. (G) The lanes indicated by the asterisks in panels (E) and (F) were run on a separate gel for clearer visualization; n = 2 (IP, immunoprecipitation; NB, not bound; IN, input).
(TIF)

S2 Fig P0-S63del protein interacts with BiP and CNX. (A) Rate of P0 proteins biosynthesis. Cells were induced for 14 hr with 100ng/ml tetracycline, pulsed and chased after 10 min. Radiolabeled P0s were immunoprecipitated with anti-HA antibody and separated in SDS-PAGE. Arrowheads indicate two additional bands that specifically co-immunoprecipitated with the misfolded P0-S63del variant. (B) Quantification of protein biosynthesis as measured by densitometric analysis. (C) Western blot anti-ubiquitin performed on lysates from HEK293 cells treated with the proteasome inhibitor PS341. Tubulin was used as loading control. (D-E) Pulse-chase experiments on HEK293 cells induced for 17 hr. Cells were pulsed with [^35^S]-methionine/cysteine for 10 min and chased for 10 min, 120 min or 120 min with PS341. First immunoprecipitation was performed against either BiP (C) or CNX (D). The CNX- and BiP-immunocomplexes were dissociated and the P0 proteins present in the complexes were re-immunoprecipitated with an anti-HA antibody. The unbound fractions (NB) of the first immunoprecipitation of lanes 2, 5 and 8 (120 min without PS341) were subjected to immunoprecipitation against the HA epitope. Samples were subjected to SDS-PAGE. Samples normalized for cell number.
(TIF)

S3 Fig Ablation of the ERAD factor Derlin-2 in Schwann cells. (A) PCR reaction on genomic DNA extracted from sciatic nerves at P5. The 600bp Der2^KO^ band appears only upon P0Cre-mediated recombination. In samples from heterozygotes Der2^SCKO/+^ animals, the 250bp Der2^+^ product derives from the wild type copy of the endogenous *Derl-2* gene. n = 2–3 mice/genotype. (B) PCR reaction on genomic DNA extracted from different tissues of Der2^SCKO^ mice at P21. (C) qRT-PCR on P28 sciatic nerve extracts to monitor Derlin-2 mRNA expression. n = 4 RT from independent pools of sciatic nerves. (D) Western blot analysis on P28 sciatic nerve lysates was performed for Derlin-2; β-Tubulin was used as loading control. One of four independent blots is shown. (E) Derlin-2 protein levels as determined by densitometric analysis. (F) qRT-PCR for OS9 mRNA on P28 sciatic nerve extracts. n = 4 RT from independent pools of sciatic nerves. (G) Western blot analysis on P28 sciatic nerve lysates for OS9 isoforms. One of four independent blots is shown. (H) OS9 protein levels as determined by densitometric analysis. (I) Western blot analysis on P28 sciatic nerve lysates for IRE1α. One of three independent blots is shown. (J) IRE1α protein levels as determined by densitometric analysis. Error bars, SEM; *P < 0,05, **P < 0,01, ***P < 0,001 by unpaired Student’s *t* test.
(TIF)

S4 Fig Derlin2 is dispensable for developmental myelination and remyelination. (A) Transverse semithin sections from WT and Der2^SCKO^ sciatic nerves at P5 and P15. n = 3–5 mice/genotype. Scale bar, 10**μ**m. (B) Sciatic nerve crush on 2 mo old WT and Der2^SCKO^ littermates. Semithin sections show crushed distal stumps (5 mm from the injury site) and contralateral control nerves 45 days after injury (T45). Yellow arrowhead indicates an example of remyelinated fiber; red arrowhead shows a degenerating fiber. Scale bar, 10μm; n = 5 mice/genotype. (C) Quantification of remyelinated and (D) degenerating fibers performed on semithin sections of crushed sciatic nerves. n = 5 nerves/genotype. (E) EM analysis reveals equal extent of remyelination in WT and Der2^SCKO^ as measured by (F) g-ratio quantitative analysis (mean g-ratio: WT control 0.64±0.003; Der2^SCKO^ control 0.65±0.003; WT crushed 0.68±0.004; Der2^SCKO^ crushed 0.67±0.006); n = 50–70 fibers per nerve, three mice per genotype; P = n.s. by one-way ANOVA with Tukey’s post hoc test. In (E), scale bar, 5**μ**m.
(TIF)

S5 Fig Derlin2 ablation worsens hypomyelination in S63del nerves but does not alter cell numbers. (A) EM images from WT, Der2^SCKO^, S63del and S63del//Der2^SCKO^ sciatic nerves at P28. Arrowheads show axons of similar diameter for myelin thickness comparison. (B) Mean g-ratio quantification (WT 0.64±0.003; Der2^SCKO^ 0.64±0.003; S63del 0.70±0.004; S63del//Der2^SCKO^ 0.72±0.003); n = 50–70 fibers per nerve, three nerves per genotype. **P < 0,01, ***P < 0,001 by one-way ANOVA with Tukey’s post hoc test. (C) Immunostaining on cryosections from P21 WT, Der2^SCKO^, S63del and S63del//Der2^SCKO^ sciatic nerves. 10 μm thick sections were stained with anti-MBP antibody to mark the endoneurial space and Hoechst dye to visualize cells nuclei. Scale bar, 100μm (D) Quantification of the endoneurial cells number/mm^2^; n = 2–3 nerves per genotype; Error bars, SEM.
(TIF)

S6 Fig ERAD is impaired in adult Der2^SCKO^ and S63del//Der2^SCKO^ nerves. (A) Western blot analysis for OS9 and IRE1α on sciatic nerves at 6 mo; one of three-four independent blots is shown. (B) OS9 and (C) IRE1α protein levels as determined by densitometry. (D) Western blot analysis for OS9 and IRE1α on quadriceps nerves lysates at 6 mo; one of three-four independent blots is shown. (E) OS9 and (F) IRE1α protein levels as determined by densitometry. Error bars, SEM; *P < 0,05, **P < 0,01, ***P <0,001 by unpaired, 2-tails, Student’s *t* test.
(TIF)

S7 Fig Measurement of ER stress/UPR levels in mutant quadriceps nerves at 6 mo. (A) Western blot analysis for BiP, GRP94 and P-eIF2**α** proteins performed on quadriceps nerves at 6 mo; β-Tubulin was used as loading control. One of four representative blots is shown. (B-D) Relative protein levels as measured by densitometry. (E) qRT-PCR for CHOP mRNA. n = 4 RT from independent pools of three nerves per genotype. *P <0,05, **P <0,01, ***P <0,001 by unpaired, 2-tails, Student’s *t* test.
(TIF)

S1 Table Table containing all the raw numerical data corresponding to Figures. (XLSX)
