# Supplementary figures and images for: Schwann cells ER-associated degradation contributes to myelin maintenance in adult nerves and limits demyelination in CMT1B mice
Source: PLoS Genet. 2019 Apr 17;15(4):e1008069. doi: 10.1371/journal.pgen.1008069 (PMC6488099; doi:10.1371/journal.pgen.1008069)

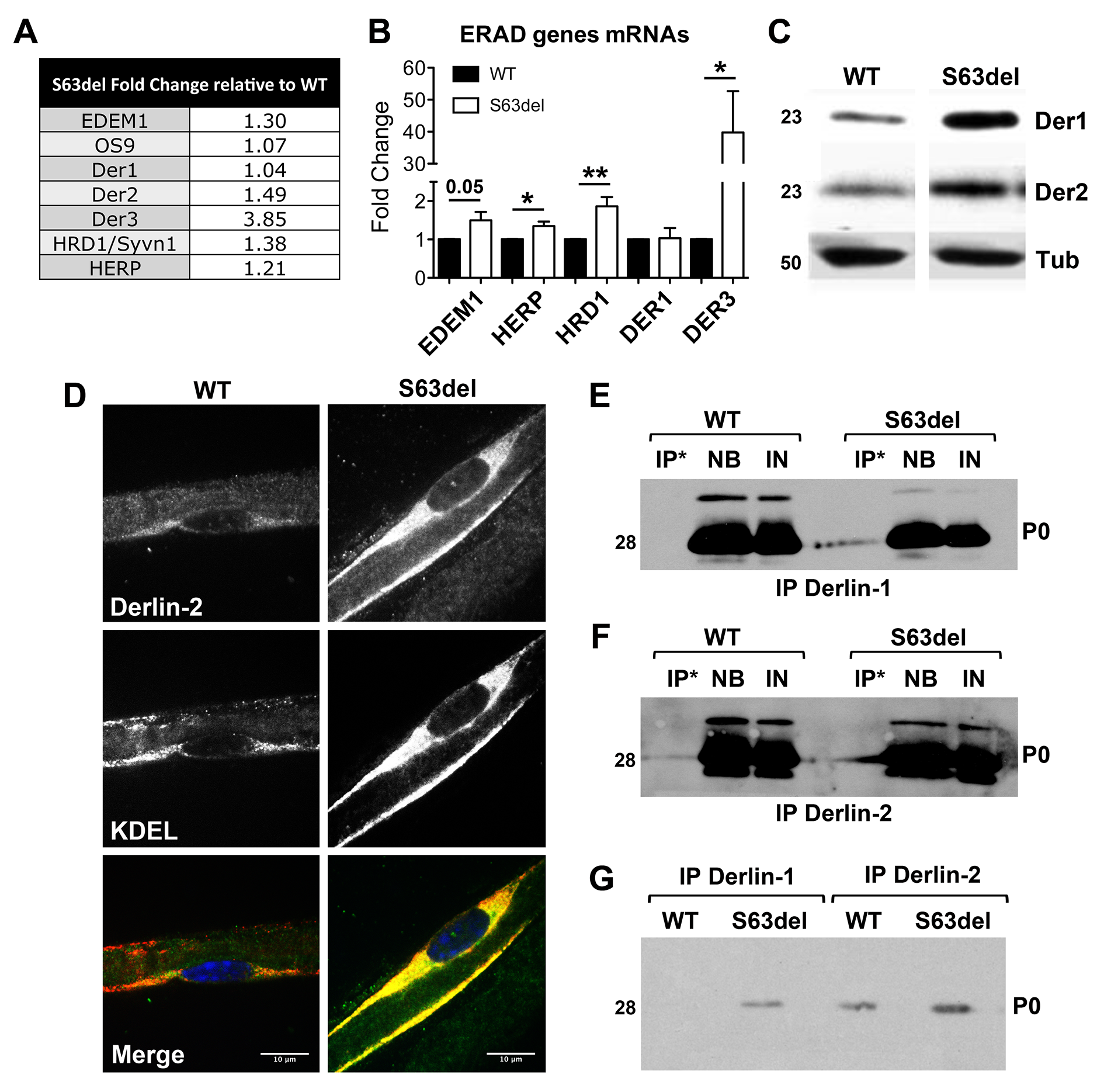

Supplement: S1 Fig — (A) Expression of ERAD genes in P28 S63del nerves relative to WT as measured by microarray analysis [11]. (B) qRT-PCR for a selection of ERAD genes on P28 S63del sciatic nerve extracts. n = 5 RT from independent pools of nerves. Error bars, SEM.; *P < 0,05, **P < 0,01 by Student’s t test. (C) Western blot on P28 sciatic nerve lysates against the ERAD members Derlin-1 and -2; β-Tubulin was used as loading control. One of four independent blots is shown. (D) Immunofluorescence for Derlin-2 (green) on P21 teased nerve fibers. In red, KDEL staining marks the Schwann cell ER; in blue, Schwann cells nuclei are visualized with Hoechst staining. Scale bar, 10μm. (E-F-G) Immunoprecipitation on WT and S63del sciatic nerve lysates with either anti-Derlin-1 (E) or anti-Derlin-2 (F) antibodies, followed by Western blot for P0. (G) The lanes indicated by the asterisks in panels (E) and (F) were run on a separate gel for clearer visualization; n = 2 (IP, immunoprecipitation; NB, not bound; IN, input). (TIF) [file pgen.1008069.s001.tif]

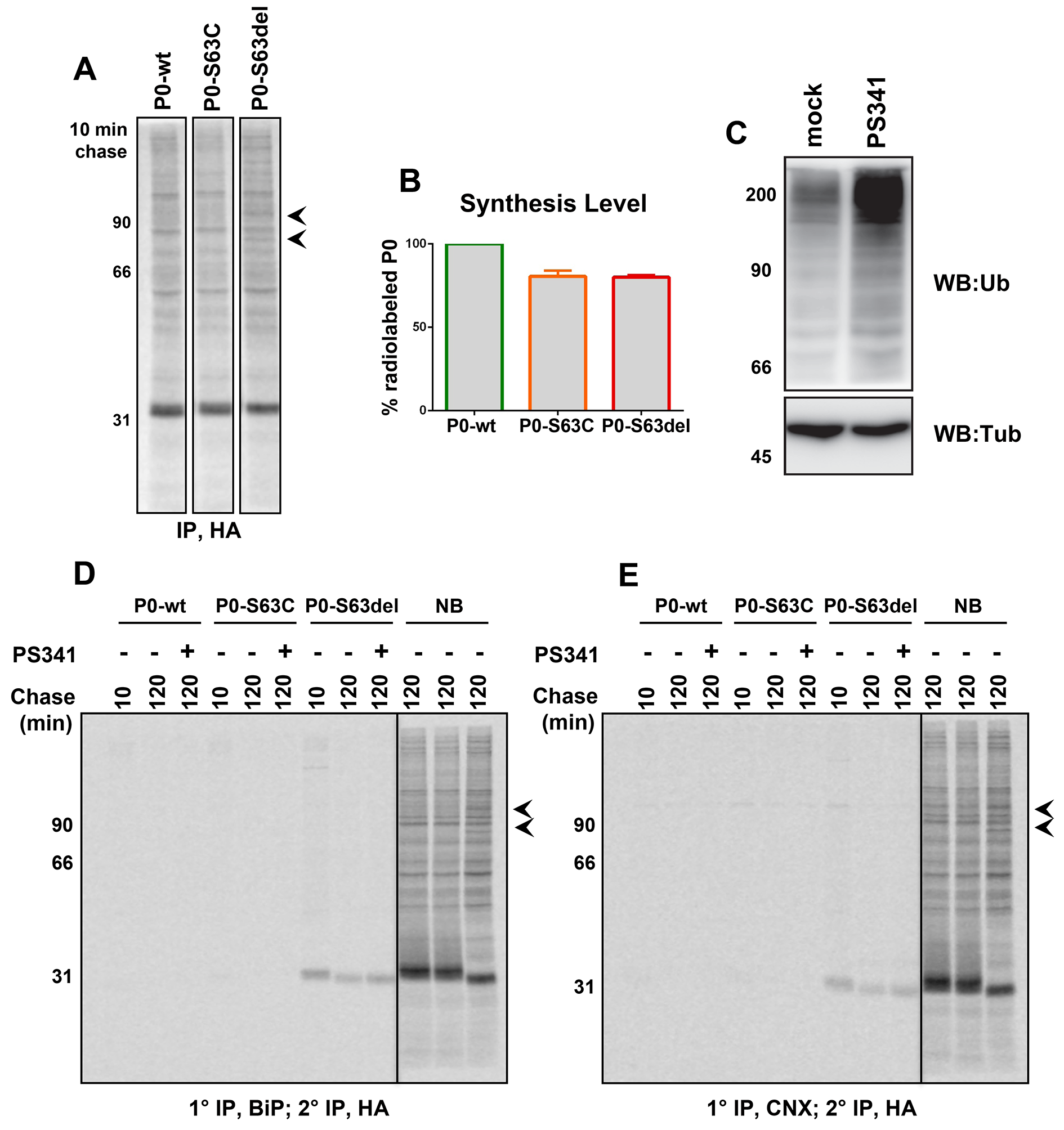

Supplement: S2 Fig — (A) Rate of P0 proteins biosynthesis. Cells were induced for 14 hr with 100ng/ml tetracycline, pulsed and chased after 10 min. Radiolabeled P0s were immunoprecipitated with anti-HA antibody and separated in SDS-PAGE. Arrowheads indicate two additional bands that specifically co-immunoprecipitated with the misfolded P0-S63del variant. (B) Quantification of protein biosynthesis as measured by densitometric analysis. (C) Western blot anti-ubiquitin performed on lysates from HEK293 cells treated with the proteasome inhibitor PS341. Tubulin was used as loading control. (D-E) Pulse-chase experiments on HEK293 cells induced for 17 hr. Cells were pulsed with [35S]-methionine/cysteine for 10 min and chased for 10 min, 120 min or 120 min with PS341. First immunoprecipitation was performed against either BiP (C) or CNX (D). The CNX- and BiP-immunocomplexes were dissociated and the P0 proteins present in the complexes were re-immunoprecipitated with an anti-HA antibody. The unbound fractions (NB) of the first immunoprecipitation of lanes 2, 5 and 8 (120 min without PS341) were subjected to immunoprecipitation against the HA epitope. Samples were subjected to SDS-PAGE. Samples normalized for cell number. (TIF) [file pgen.1008069.s002.tif]

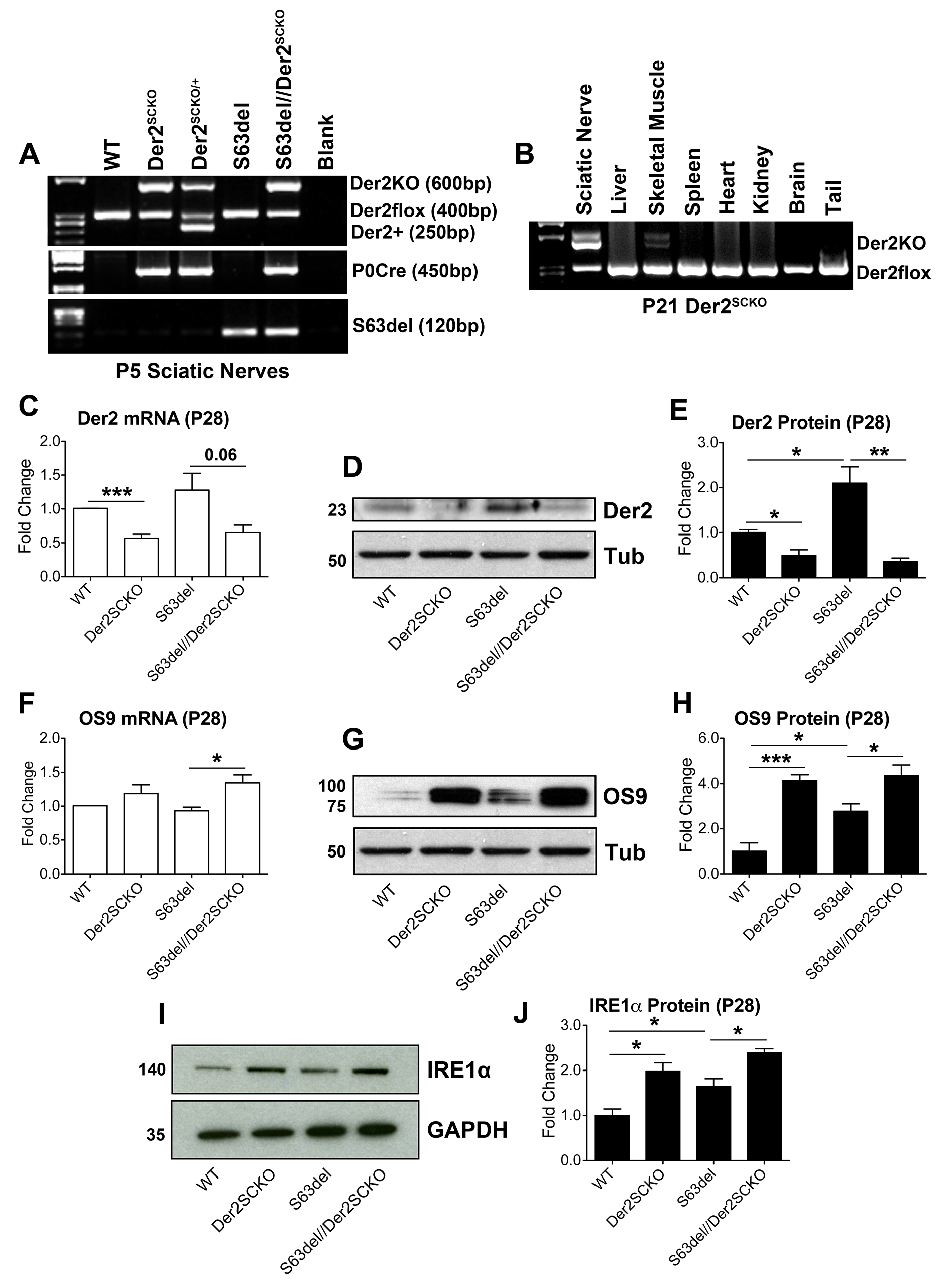

Supplement: S3 Fig — (A) PCR reaction on genomic DNA extracted from sciatic nerves at P5. The 600bp Der2KO band appears only upon P0Cre-mediated recombination. In samples from heterozygotes Der2SCKO/+ animals, the 250bp Der2+ product derives from the wild type copy of the endogenous Derl-2 gene. n = 2–3 mice/genotype. (B) PCR reaction on genomic DNA extracted from different tissues of Der2SCKO mice at P21. (C) qRT-PCR on P28 sciatic nerve extracts to monitor Derlin-2 mRNA expression. n = 4 RT from independent pools of sciatic nerves. (D) Western blot analysis on P28 sciatic nerve lysates was performed for Derlin-2; β-Tubulin was used as loading control. One of four independent blots is shown. (E) Derlin-2 protein levels as determined by densitometric analysis. (F) qRT-PCR for OS9 mRNA on P28 sciatic nerve extracts. n = 4 RT from independent pools of sciatic nerves. (G) Western blot analysis on P28 sciatic nerve lysates for OS9 isoforms. One of four independent blots is shown. (H) OS9 protein levels as determined by densitometric analysis. (I) Western blot analysis on P28 sciatic nerve lysates for IRE1α. One of three independent blots is shown. (J) IRE1α protein levels as determined by densitometric analysis. Error bars, SEM; *P < 0,05, **P < 0,01, ***P < 0,001 by unpaired Student’s t test. (TIF) [file pgen.1008069.s003.tif]

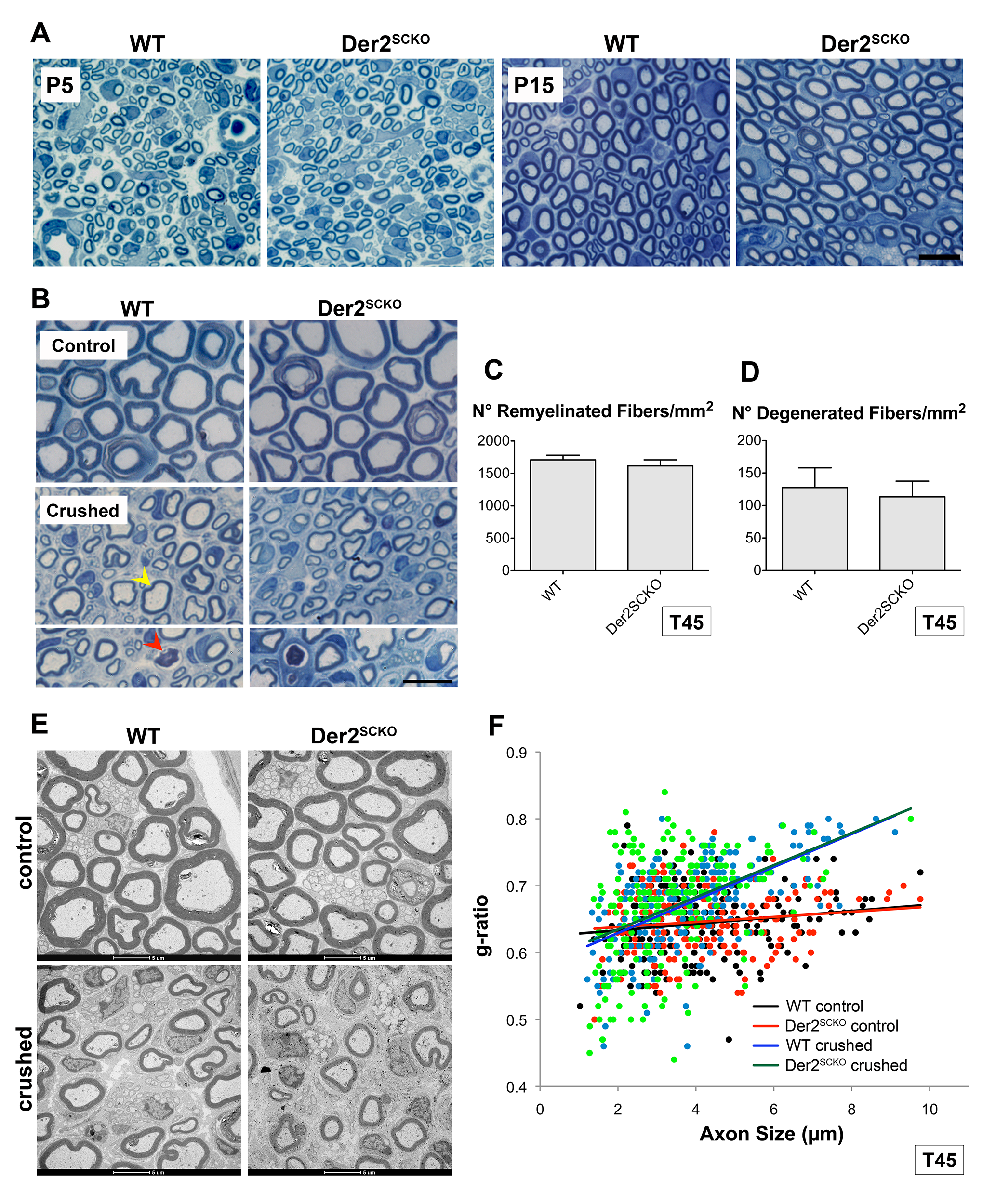

Supplement: S4 Fig — (A) Transverse semithin sections from WT and Der2SCKO sciatic nerves at P5 and P15. n = 3–5 mice/genotype. Scale bar, 10μm. (B) Sciatic nerve crush on 2 mo old WT and Der2SCKO littermates. Semithin sections show crushed distal stumps (5 mm from the injury site) and contralateral control nerves 45 days after injury (T45). Yellow arrowhead indicates an example of remyelinated fiber; red arrowhead shows a degenerating fiber. Scale bar, 10μm; n = 5 mice/genotype. (C) Quantification of remyelinated and (D) degenerating fibers performed on semithin sections of crushed sciatic nerves. n = 5 nerves/genotype. (E) EM analysis reveals equal extent of remyelination in WT and Der2SCKO as measured by (F) g-ratio quantitative analysis (mean g-ratio: WT control 0.64±0.003; Der2SCKO control 0.65±0.003; WT crushed 0.68±0.004; Der2SCKO crushed 0.67±0.006); n = 50–70 fibers per nerve, three mice per genotype; P = n.s. by one-way ANOVA with Tukey’s post hoc test. In (E), scale bar, 5μm. (TIF) [file pgen.1008069.s004.tif]

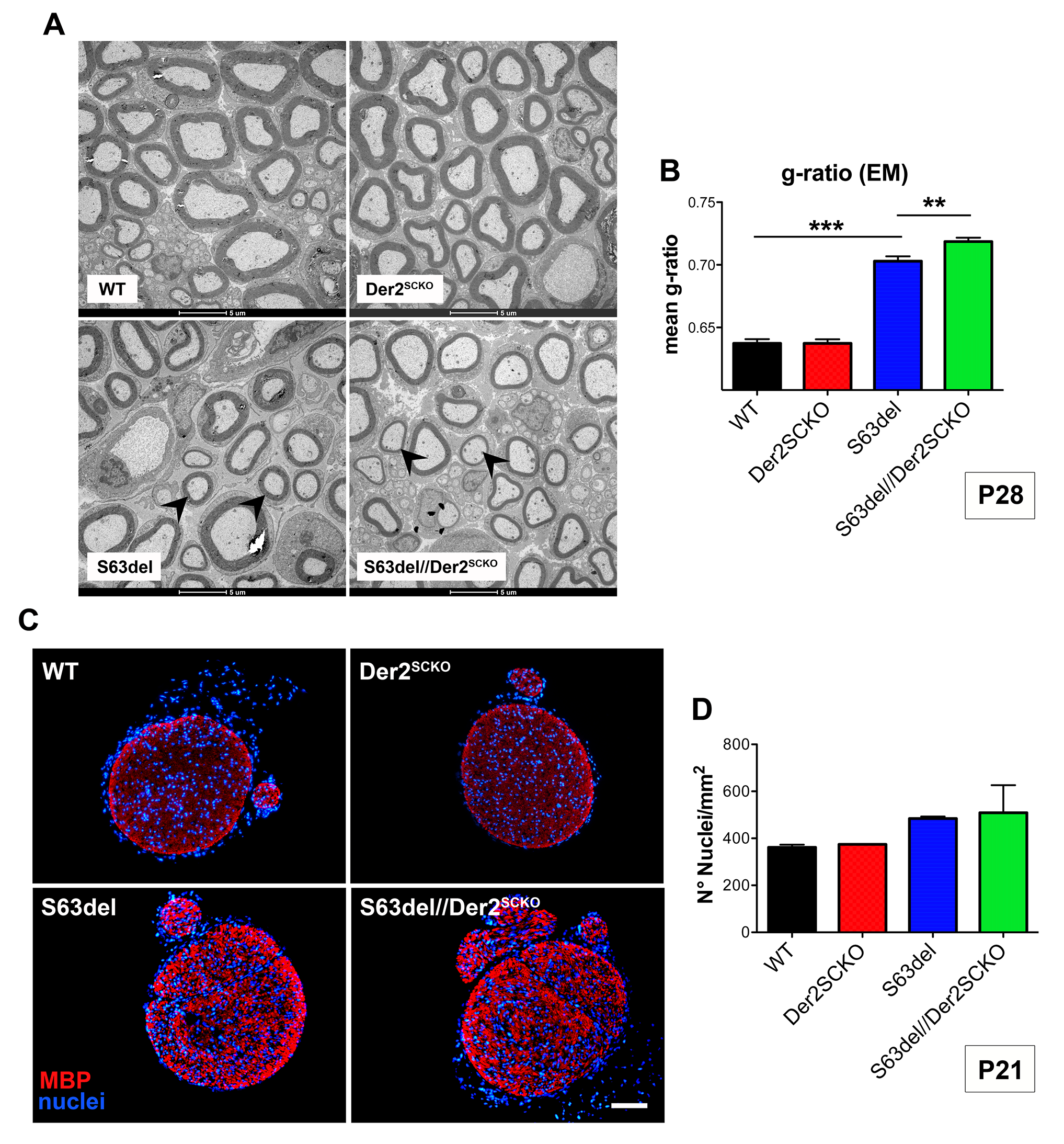

Supplement: S5 Fig — (A) EM images from WT, Der2SCKO, S63del and S63del//Der2SCKO sciatic nerves at P28. Arrowheads show axons of similar diameter for myelin thickness comparison. (B) Mean g-ratio quantification (WT 0.64±0.003; Der2SCKO 0.64±0.003; S63del 0.70±0.004; S63del//Der2SCKO 0.72±0.003); n = 50–70 fibers per nerve, three nerves per genotype. **P < 0,01, ***P < 0,001 by one-way ANOVA with Tukey’s post hoc test. (C) Immunostaining on cryosections from P21 WT, Der2SCKO, S63del and S63del//Der2SCKO sciatic nerves. 10 μm thick sections were stained with anti-MBP antibody to mark the endoneurial space and Hoechst dye to visualize cells nuclei. Scale bar, 100μm (D) Quantification of the endoneurial cells number/mm2; n = 2–3 nerves per genotype; Error bars, SEM. (TIF) [file pgen.1008069.s005.tif]

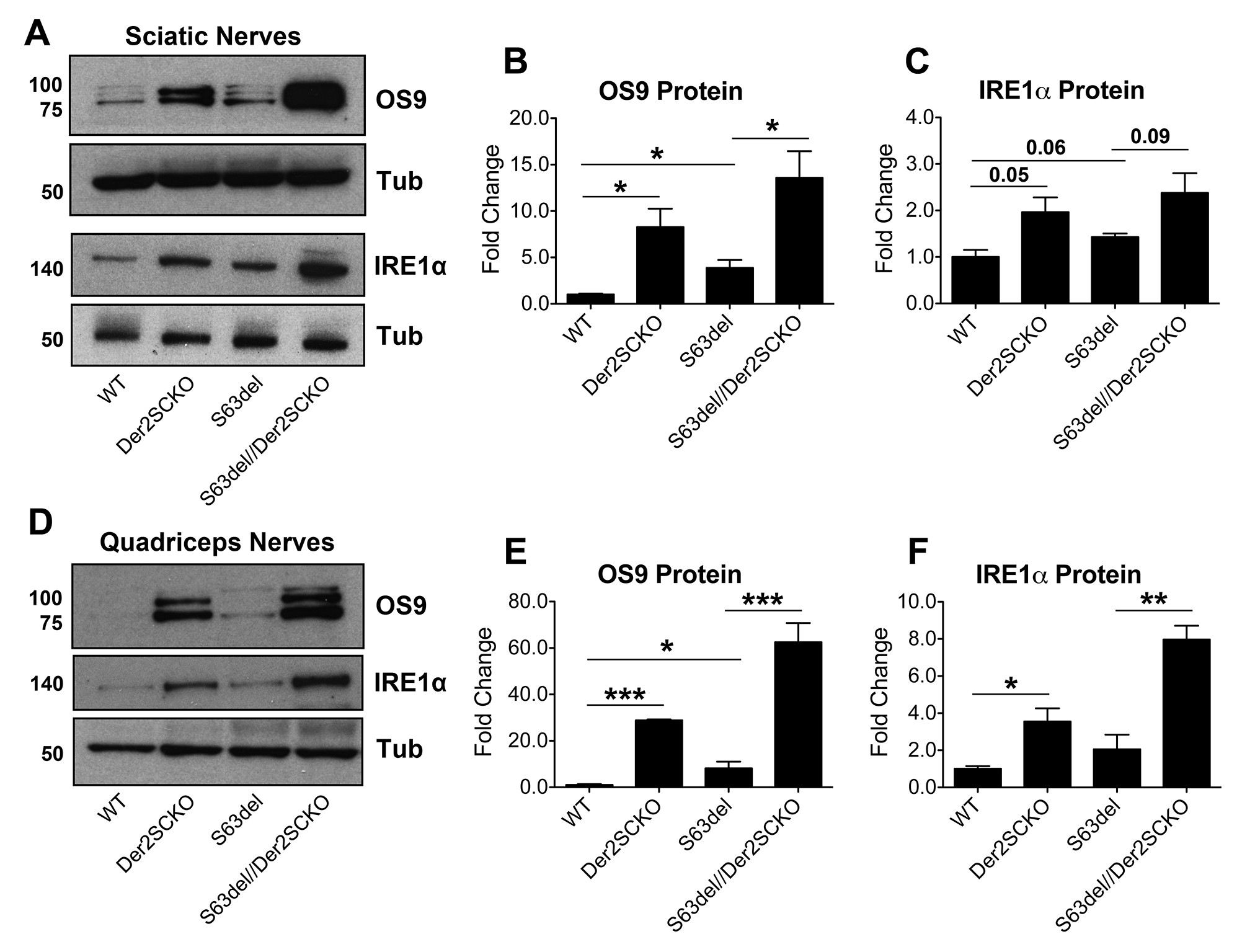

Supplement: S6 Fig — (A) Western blot analysis for OS9 and IRE1α on sciatic nerves at 6 mo; one of three-four independent blots is shown. (B) OS9 and (C) IRE1α protein levels as determined by densitometry. (D) Western blot analysis for OS9 and IRE1α on quadriceps nerves lysates at 6 mo; one of three-four independent blots is shown. (E) OS9 and (F) IRE1α protein levels as determined by densitometry. Error bars, SEM; *P < 0,05, **P < 0,01, ***P <0,001 by unpaired, 2-tails, Student’s t test. (TIF) [file pgen.1008069.s006.tif]

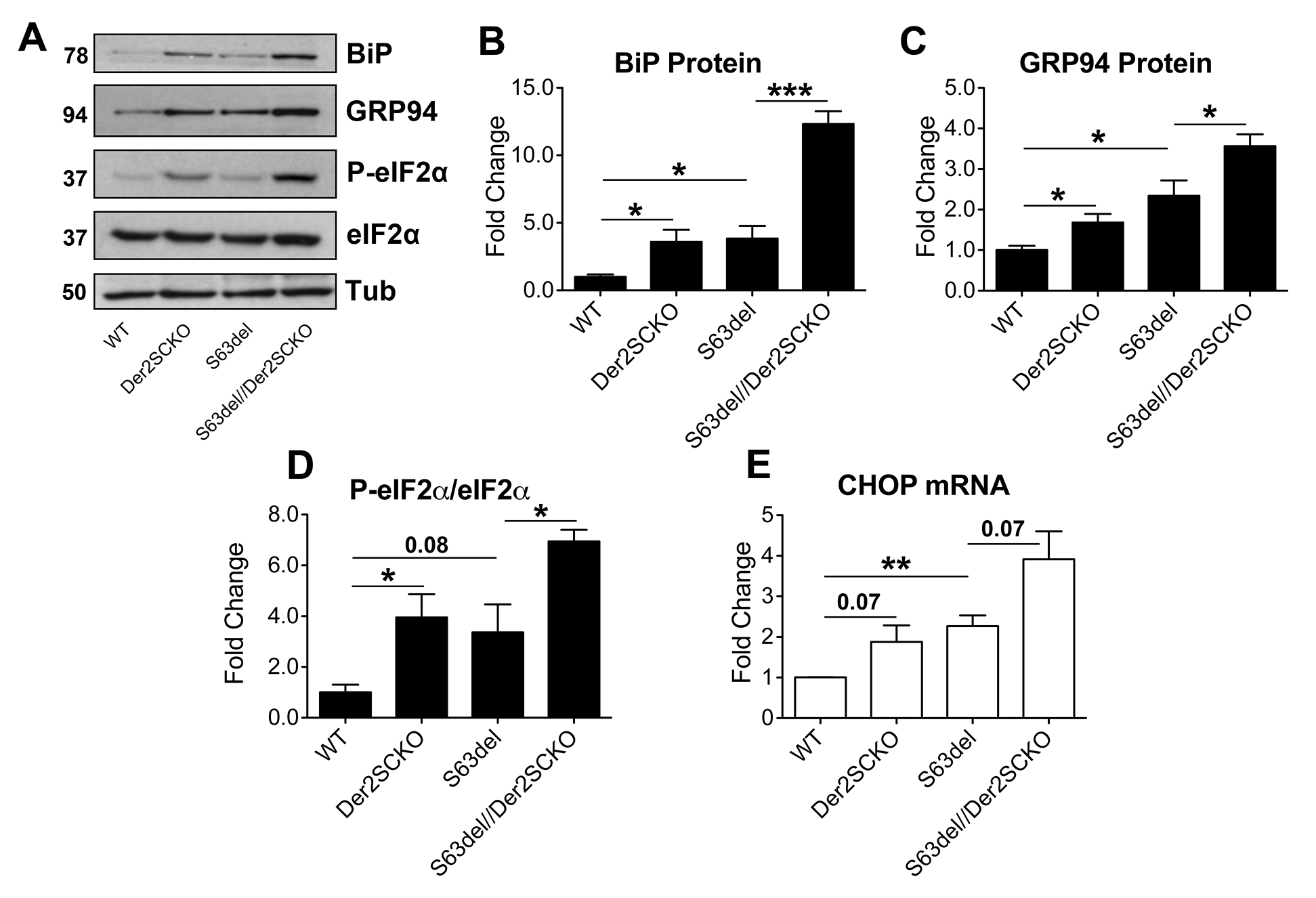

Supplement: S7 Fig — (A) Western blot analysis for BiP, GRP94 and P-eIF2α proteins performed on quadriceps nerves at 6 mo; β-Tubulin was used as loading control. One of four representative blots is shown. (B-D) Relative protein levels as measured by densitometry. (E) qRT-PCR for CHOP mRNA. n = 4 RT from independent pools of three nerves per genotype. *P <0,05, **P <0,01, ***P <0,001 by unpaired, 2-tails, Student’s t test. (TIF) [file pgen.1008069.s007.tif]
